# A nanodrug system overexpressed circRNA_0001805 alleviates nonalcoholic fatty liver disease via miR-106a-5p/miR-320a and ABCA1/CPT1 axis

**DOI:** 10.1186/s12951-021-01108-8

**Published:** 2021-11-17

**Authors:** Jian Li, Jing Qi, Yishu Tang, Huaizheng Liu, Kefu Zhou, Zheren Dai, Lehong Yuan, Chuanzheng Sun

**Affiliations:** 1 Department of Blood Transfusion, Third Xiangya Hospital, Central South University, Changsha, 410013 Hunan China; 2 Department of Emergency, Third Xiangya Hospital, Central South University, Changsha, 410013 Hunan People’s Republic of China

**Keywords:** circRNA_0001805, ABCA1, CPT1, Nanomedicine system, NAFLD

## Abstract

Our study aimed to explore the function of circRNA_0001805 in the pathogenesis of NAFLD and the underlying mechanism. A nanodrug system (GA-RM/GZ/PL) was constructed to overexpress circRNA_0001805 specifically in hepatocytes for the treatment of NAFLD. Fat droplet accumulation in cultured cells and mouse hepatic tissues was detected using Oil Red O or H&E staining. The relative expression of circRNAs, genes associated with lipogenesis was quantified by qRT-PCR. Interactions between circRNA_0001805 and miR-106a-5p/miR-320a, between miR-106a-5p/miR-320a and ABCA1/CPT1 were confirmed by dual-luciferase reporter assay. A novel metalorganic framework nanocarrier (GZ) was prepared from glycyrrhizic acid and zinc ions (Zn^2+^), and this nanocarrier was loaded with the circRNA_0001805 plasmid to construct a nanocore (GZ/PL). Then, this GZ/PL was coated with a galactose-modified RBC membrane (GA-RM) to generate GA-RM/GZ/PL. CircRNA_0001805 expression was downregulated in FFA-challenged primary hepatocytes, HFD-fed mice and NAFLD patients. Overexpressed circRNA_0001805 attenuated NAFLD development by suppressing lipid metabolism disorder and inflammation. CircRNA_0001805 targeted miR-106a-5p/miR-320a, which served as an upstream inhibitor of ABCA1/CPT1 and collaboratively regulated NAFLD progression. GA-RM/GZ/PL targeted hepatocytes, overexpressed circRNA_0001805, released glycyrrhizic acid to reduce the accumulation of lipids in the liver and played a synergistic role against NAFLD-induced lipid metabolism disorder.

**Supplementary Information:**

The online version contains supplementary material available at 10.1186/s12951-021-01108-8.

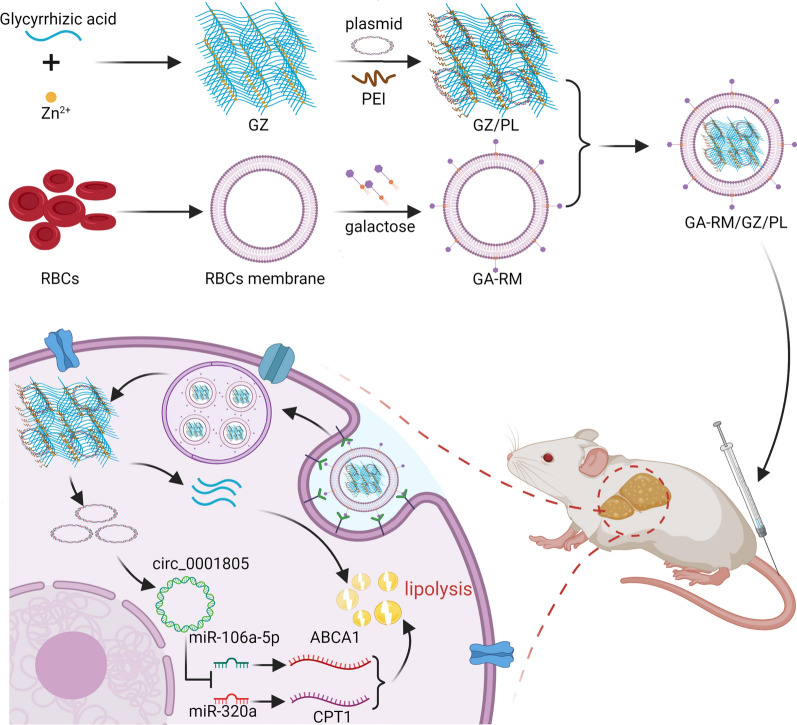

## Introduction

Nonalcoholic fatty liver disease (NAFLD) is one of the most common chronic hepatic diseases, and more than 20% of the world’s population is affected [1]. NAFLD is a disorder characterized by excess fat deposition in the liver that is not caused by alcohol consumption, and NAFLD may further progress to nonalcoholic steatohepatitis (NASH), cirrhosis and hepatocellular carcinoma (HCC) due to chronic inflammation [2]. NAFLD is closely related with overweight- or obesity-induced insulin resistance via complex mechanisms, and its prevalence among type 2 diabetes mellitus (T2DM) patients is estimated to be as high as 70% [3]. Due to rapid changes in urbanized lifestyles, such as unhealthy dietary habits, and the consequent dramatic increase in the incidence of T2DM in recent decades, NAFLD has emerged as a growing yet critical global health problem [4, 5]. However, there are currently no effective medical treatments for NAFLD patients, and the development of optimal pharmacological interventions is limited mainly by our incomplete understanding of its heterogeneous pathogenesis.

NF-κB is a multifunctional nucleoprotein transcription factor. The most common form of NF-κB in cells is a heterodimer composed of P65 and P50 [6]. In its resting state, NF-κB binds to IκB and remains in the cytoplasm in an inactive form [7]. NAFLD is mainly characterized by the accumulation of fatty acids and triglycerides in the liver, which increase the infiltration of macrophages and the expression level of inflammatory factors, such as TNF-α and IL-6 [8]. After activation by inflammatory factors (TNF-α and IL-6), IκB is phosphorylated by IK kinase (IKKβ) and dissociates from NF-κB, thus disinhibiting its activity [9]. NF-κB enters the nucleus and effectively induces the expression of inflammatory cytokines (TNF-α and IL-6), which play a cascading role in amplifying the inflammatory response [10]. Thus, the NF-κB signaling pathway plays a vital role in regulating NAFLD progression and represents a promising therapeutic target [11].

Carnitine palmitoyl transferase 1 (CPT1) is the channel for fatty acids to enter the mitochondrial matrix and is the main factor that controls 80% of fatty acid β-oxidation in hepatic mitochondria [12]. CPT1 expression is significantly inhibited during liver injury and fat accumulation, and CPT1 knockdown in mice results in severe liver fat accumulation and inflammation [13]. The decrease of mitochondrial β-oxidation caused by reduced CPT1 expression may be a key event in the pathogenesis of hepatic steatosis in mice [14]. ATP-binding cassette transporter A1 (ABCA1) is a membrane protein that uses ATP as energy for the transport of molecules, such as cholesterol and phospholipids, and plays an important role in the initial steps of cholesterol reversal transport (RCT) and high-density lipoprotein (HDL) generation [15, 16]. In a mouse model of NAFLD, liver-specific ABCA1 inactivation resulted in a decrease in plasma HDL levels and cholesterol efflux from hepatocytes [17]. Disorders in fatty acid β-oxidation and cholesterol outflow in liver cells lead to hepatic lipid aggregation and cause a chronic inflammatory response [18].

Circular RNAs (circRNAs) are noncoding RNAs with covalently linked 3′ and 5′ ends [19]. Although previously categorized as nonfunctional splicing byproducts, ample evidence suggests that circRNAs play an important role in gene transcription by forming complexes with RNA-binding proteins (RBPs) or miRNAs, and circRNAs have been implicated in a variety of human diseases [20]. Notably, Guo et al. identified 357 circRNAs that were differentially expressed in a hepatic steatosis cellular model, among which 154 were upregulated and 203 were downregulated [21]. In this study, 84 upregulated and 100 downregulated circRNAs were significantly differentially expressed in NAFLD liver tissues compared with normal liver tissues according to a circRNA microarray. CircRNA_0001805 expression was found to be downregulated in both this study and Guo’s study [21]. Although some research has been reported, there are very few studies on the role of circRNAs during the progression of NAFLD and the underlying mechanism [22, 23].

Traditional gene vectors (such as viral vectors and liposomes) have limitations, such as high immunogenicity, high cytotoxicity and single functions [24]. Nanotechnology is reshaping healthcare strategies [25]. Compared with traditional drugs, nanodrugs can effectively target diseased tissues and effectively control drug release [26–28]. Nanotechnology has been used to successfully deliver siRNAs that target tumor cells and achieve therapeutic effects [29]. Metal–organic frameworks (MOFs) are a class of highly ordered crystalline porous coordination polymers with the advantages of high specific surface area and porosity, adjustable pore size and easy chemical modification, which have become a promising class of nanodrug carriers [30]. Traditional nanomaterials are easy to be identified and cleared in vivo, resulting in short circulation time and limited application. In recent years, cell membrane camouflage technology endows nanocore with good stability and immune escape function, and has become an emerging strategy [31]. On this basis, this project aimed to prepare a specific hepatocellular-targeted gene vector for the treatment of NAFLD through nanotechnology.

**Fig. 1:**
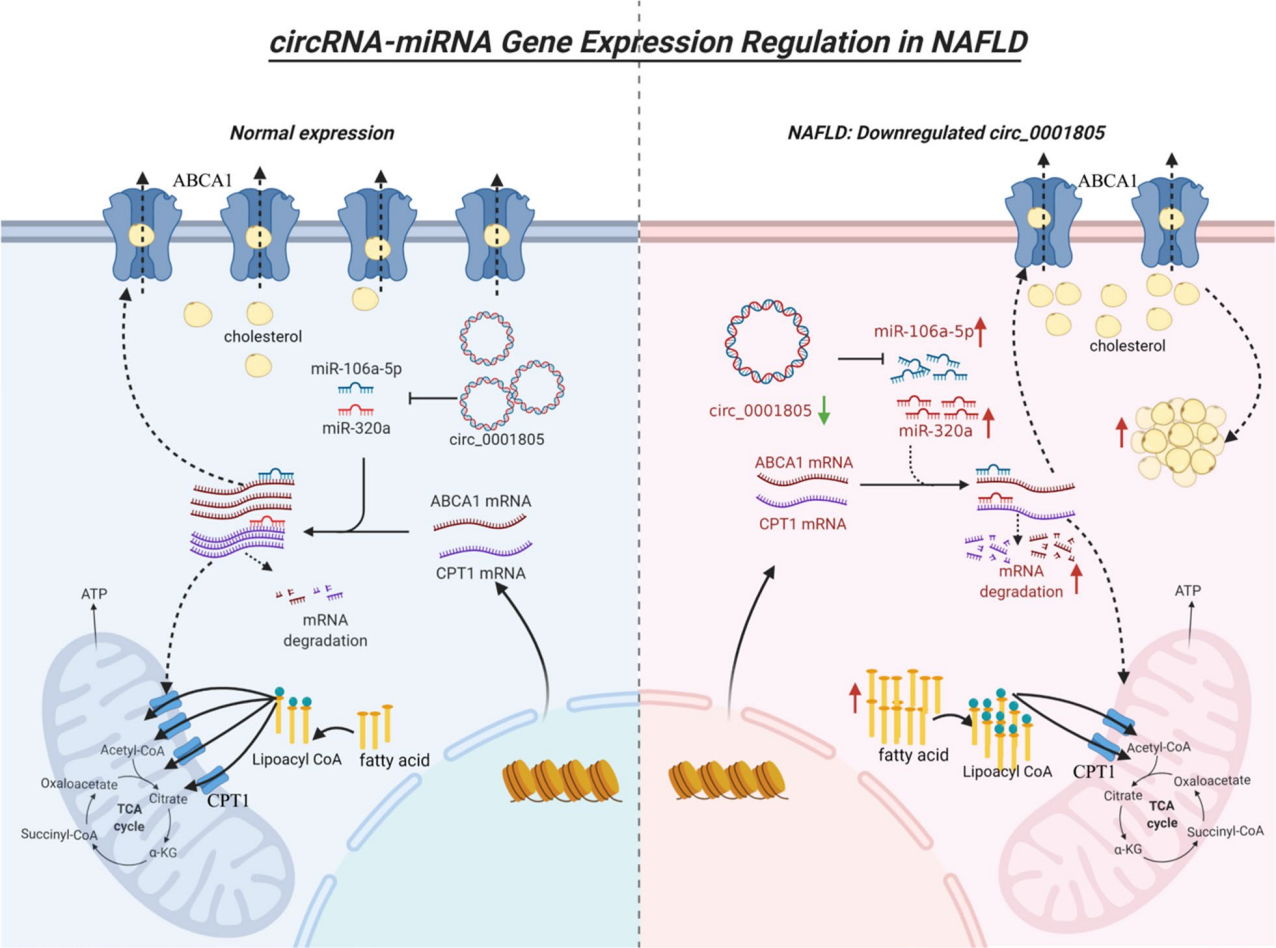
circRNA-miRNA gene expression regulation in NAFLD

In this study, we performed in vitro and in vivo assays and demonstrated that circRNA_0001805 expression was decreased in NAFLD cellular and animal models as well as in hepatic tissues obtained from NAFLD patients. As shown in Fig. 1, we predicted that miR-106a-5p and miR-320a are potential binding targets of circRNA_0001805, and miR-106a-5p and miR-320a were also found to be putative binding partners of ABCA1 and CPT1. Herein, we validated the direct binding of circRNA_0001805 to miR-106a-5p and miR-320a and explored the interactions among circRNA_0001805, miR-106a-5p, miR-320a, ABCA1 and CPT1 during the progression of NAFLD. We showed that circRNA_0001805 exerts protective effects in NAFLD cellular and animal models by modulating the NF-κB signaling axis via miR-106a-5p, miR-320a/ABCA1 and CPT1.

**Fig. 2:**
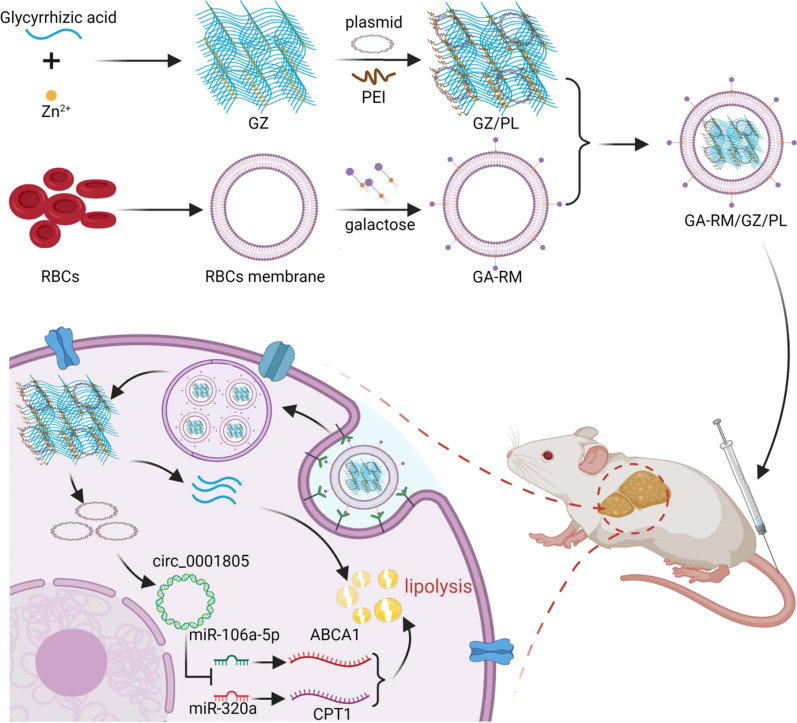
Schematic diagram of GA-RM/GZ/PL construction and targeted application in NAFLD

Furthermore, as shown in Fig. 2, a novel metalorganic framework nanocarrier (GZ) was successfully prepared from glycyrrhizic acid and zinc ions (Zn^2+^), and this nanocarrier was loaded with a circRNA_0001805 plasmid to generate a nanocore (GZ/PL). Finally, a nanomedicine system (GA-RM/GZ/PL) was prepared by coating the GZ/PL with a galactose-modified RBC membrane (GA-RM) to enable the targeting of the liver and immune escape. The results showed that GA-RM/GZ/PL had good biocompatibility, could efficiently carry plasmid RNA, and had high transfection efficiency. In addition, glycyrrhizic acid itself inhibits lipid deposition and exerts anti-inflammatory and detoxification effects in NAFLD [32]. Therefore, we speculated that GA-RM/GZ/PL could cooperate to play a synergistic role in the treatment of NAFLD.

## Materials and methods

### Materials

Glycyrrhizic acid (DG0006) was obtained from Chengdu DeSiTe Biological Technology Co., Ltd (China). Zinc sulfate (A602906) and galactose (A600215) were purchased from Sangon Biotech (China). PEI (E8420), Hemotoxylin and Eosin staining kit (G1120) and RIPA lysis buffer (R0010) were purchased from Solarbio Life Science (China). DSPE-PEG 2000 was produced by Xi'an Ruixi Biological Technology Co., Ltd. (China). Penicillin–streptomycin, fetal bovine serum (FBS) and Dulbecco's modified Eagle’s medium (DMEM) were produced by Gibco (USA). Free fatty acids (1170460001), palmitic acid (P0500) and Oil Red O (O0625) were obtained from Sigma (USA). p-IKKβ (Abcam, ab59195, 1:1000), IKKβ (Abcam, ab124957, 1:1000), p-p65 (ab86299), p65 (ab16502), TNF-α (ab183218), IL-6 (ab259341), ABCA1 (ab66217), CPT1 (ab234111) primary antibodies and horseradish peroxidase (HRP)-labeled secondary antibodies were obtained from Abcam (USA).

### Human liver specimens and ethic statement

Fresh human liver tissues were collected from NAFLD patients (N = 25) who underwent liver transplantation at the Third Xiangya Hospital from 2016 to 2018. *N* = *9* cases of control samples were obtained from normal liver donors. Written informed consent was collected from all participants. The experimental procedures involving human liver samples in this study were approved by Central South University ethics committee. All liver specimens were flash frozen in liquid nitrogen and further experiments were conducted in strict accordance with the Helsinki Declaration.

### Primary human hepatocytes and cell culture

Primary Human Hepatocytes with high viability were obtained from Corning (MTOXH1005) and maintained according to manufacturer’s instructions. The recovered primary hepatocytes were cultured in DMEM (Gibco, USA), which was supplemented with 10% fetal bovine serum (FBS, Gibco, USA), 100 U/ml penicillin and 0.1 mg/ml streptomycin (Sigma, USA), and seeded on collagen-coated 6-well plate at a density of 2 × 10^6^ cells per well. In order to generate a cellular NAFLD model, primary human hepatocytes were incubated in complete DMEM containing 1 mM free fatty acids (FFA) consisting 0.667 mM palmitic acid (Sigma, USA) and 0.333 mM oleate acid for an additional 48 h, and lipid accumulation was monitored under microscope.

### In vivo* NAFLD model*

All the animal experiment schemes were approved by the Animal Use and Care Committee of Central South University. Male C57BL/6 (20 g) mice were obtained from Hunan SJA Laboratory Animal Co., Ltd (Hunan, China) and allowed to acclimate for a week with unlimited access to fresh water and food before each experiment. To generate an NAFLD animal model, mice were fed with the high fat diet (HFD) (60% Kcal Fat) for up to eight weeks. The weight of each mouse was monitored every four days. Mice fed with a normal chow diet (NCD) were used as negative control. For in vivo overexpression of circRNA_0001805, mice fed by HFD were injected with 10^9^ PFU of adenovirus harboring circRNA_0001805 in 100 µl PBS or empty adenoviruses through tail vein. Animals were sacrificed after eight weeks and liver tissues were carefully excised for weight measurement.

### Oil Red O staining

Lipid staining of cultured cells and animal liver tissues was performed using Oil Red O (Sigma, USA). Briefly, cells were cultured on glass coverslips in a 6-well plate and then fixed with 4% paraformaldehyde at 4 ℃ for 15 min. Next, cells were rinsed with PBS and incubated with Oil Red O reagent for 15 min. Finally, Oil Red O was removed and the coverslips were washed three times with distilled water. For staining of the tissues, frozen left liver lobe was cut into 4 µm slices and affixed to microscope slides. The slides were washed with tap water and then 60% isopropanol, followed by staining with Oil Red O. Images of cell samples and tissue sections were acquired with a light microscope (Zeiss, Germany).

### Metabolic assays

Total cholesterol (TC) and triglyceride (TG) were detected with commercial kits (Solarbio, Beijing, China) according to the manufacturer’s protocols.

### Hemotoxylin and Eosin (H&E) staining

Mice liver tissues were fixed with 4% paraformaldehyde for 12 h and embedded in paraffin. Tissues were cut into 4 µm slices and affixed to a microscope slide. Next, slides were placed in a slide holder and paraffin was dissolved by two changes of xylene, followed by re-hydration with sequential changes of 100, 95, and 70% ethanol. For the staining of nuclei, hemotoxylin was applied to the slides and incubated at room temperature for 8 min. After thoroughly washing with tap water and differentiation with 1% acid ethanol, tissue samples were stained with eosin for 30 s. Images of tissue samples were acquired with a light microscope (Zeiss, Germany).

### Western blot analysis (WB)

Total protein was extracted from cultured cells and liver tissues by using ice cold RIPA lysis buffer. The protein concentration was determined by BCA protein assay kit (Solarbio, Beijing, China). 20 μg of protein was ran on a 10% SDS-PAGE gel and transferred to a PVDF (Millipore, USA) membrane. GAPDH was used as a control and non-specific bindings were blocked by 5% BSA for 40 min at room temperature. Incubates the specific primary antibodies against p-IKKβ (Abcam, ab59195, 1:1000), IKKβ (Abcam, ab124957, 1:1000), p-p65 (Abcam, ab86299, 1:1000 dilution), p65 (Abcam, ab16502, 1:1000 dilution), TNF-α (Abcam, ab183218, 1:1000 dilution), IL-6 (Abcam, ab259341, 1:1000 dilution), ABCA1 (Abcam, ab66217, 1:1000), CPT1 (Abcam, ab234111, 1:1000) at 4 ℃ overnight. After extensively washing with 100 µM PBST, peroxidase-conjugated secondary antibody (Abcam, ab205718, 1:5000) was applied to the membranes and incubated for 2 h at room temperature. Protein bands were detected by using ECL substrates (Thermal Fisher, Rockford, USA) and visualized under a ChemiDoc XRS + imager (Bio-Rad, Hercules, USA).

### Quantitative RT-PCR (qRT-PCR)

Total RNA was acquired from snap-frozen hepatic tissues and cells with TRIzol reagent (Dongsheng Biotech, China). The purity and concentration of the RNA samples were determined by UV spectroscopy at wavelengths of 260 and 280 nm. qRT-PCR was performed by a One Step SYBR PrimeScript RT-PCR Kit (Takara, Dalian, China). Briefly, first-strand cDNA was synthesized using PrimerScript Reverse Transcriptase. The amplified PCR products were measured by using SYBR Green. The relative expression of each target gene was normalized to that of the internal control U6 by using the 2-ΔΔCT method. The primer sequences used for qRT-PCR were commercially synthesized by Shengong Co., Ltd. (Shanghai, China) and are listed in Additional file 1: Table S1.

### shRNA and adenoviral vector transfection

cDNA of circRNA_0001805 and short hairpin RNAs (shRNA) specifically targeting CPT1 and ABCA1 were commercially synthesized (GenePharm, Shanghai, China) and subcloned to the shuttle vector pShuttle and linearized into viral backbone vector pAdEasy-1 (Agilent Technologies, Beijing, China). The selected recombinant construct was amplified using XL10-Gold strain and applied to transfect HEK293 cells using Lipofectamine 2000 (Invitrogen, USA). The adenoviruses were purified and plaque assay was conducted to determine the viral titer (10^9^ PFU/mL). For stable expression or knockdown, primary hepatocytes were transfected with generated adenoviruses above for 24 h and subjected to the subsequent analysis. Empty viruses served as a negative control (NC).

### Luciferase reporter assay

Potential binding sites between circRNA_0001805 and miR-106a-5p/miR-320a, miR-106a-5p/miR-320a and ABCA1/CPT1 were analyzed and predicted using the web servers TargetScan and Starbase. CircRNA_0001805 (WT circRNA_0001805), 3′-UTR of wild type ABCA1 and CPT1 (WT ABCA1 and WT CPT1) were subcloned into psiCHECK2 dual luciferase vector (Promega Biotech, WI, USA). The mutations of the potential binding sites (MUT circRNA_0001805, MUT ABCA1 and MUT CPT1) were constructed by QuickChange II Site-Directed Mutagenesis Kit (Agilent Technologies, Beijing, China). For validation of the interaction between circRNA_0001805 and miR-106a-5p/miR-320a, HEK293T cells were co-transfected with WT circRNA_0001805 or MUT circRNA_0001805 and miR-106a-5p, miR-320a or control mimics using Lipofectamine 3000 (Invitrogen, Shanghai, China). For validation of the interaction between miR-106a-5p/miR-320a and ABCA1/CPT1, HEK293T cells were co-transfected with WT ABCA1, WT CPT1, MUT ABCA1 or MUT CPT1 and miR-106a-5p, miR-320a or control mimics. Cells were lysed after 48 h and luciferase activities were measured with Dual-Luciferase Reporter Assay System vector (Promega, USA). Relative luciferase activity was calculated as the ratio of Renilla luciferase activity to firefly luciferase activity.

### Preparation of GA-RM/GZ/PL

Glycyrrhizic acid (0.6 g) and Zn(NO3)_2_ (0.2 g) were dissolved in 10 ml of double distilled water. The glycyrrhizic acid solution was added slowly to the Zn(NO3)_2_ and stirred for 24 h. The supernatant was collected by centrifugation and discarded, and the pellet was washed with distilled water for 3 times to obtain GZ. GZ was modified with polyethylene imine (PEI), and 1 mg of circRNA_0001805 plasmid was added to obtain GZ/PL. Whole blood was collected from mice into EDTA-coated tubes and centrifuged at 4000 rpm for 10 min, and the remaining RBCs were lysed with 0.25 × PBS for 10 min. The supernatant was collected by centrifugation and discarded, and the sample was treated with 0.25 × PBS hypoosmosis solution 2 times. The remaining cell membrane was extracted by centrifugation, and nanoscale RBC membrane (RM) vesicles were obtained by ultrasound. Galactose (2 mg), DSPE-PEG2000 (10 mg), EDC (1.5 mg) and NHS (0.8 mg) were dissolved in 0.5 ml PBS and stirred overnight at room temperature. The free EDC, NHS and galactose were dialyzed in a dialysis bag, and then, the sample was added to the prepared RM to synthesize a galactose-modified erythrocyte membrane (GA-RM). GA-RM and GZ/PL were mixed with ultrasonication (42 kHz, 100 W) for 2 min to obtain GA-RM/GZ/PL. The content of plasmid DNA in the dialysate was detected at 260 nm. The content of galactose in the dialysate was determined by Galactose Assay Kit (Ab83382, Abcam, USA). Finally, the encapsulation efficiency (EE) and loading capacity (LC) of plasmid DNA and galactose were calculated according to the following formula:

EE = weight of drug into nanoparticles/weight of the feeding drug × 100%

LC = weight of drug into nanoparticles/(weight of nanoparticles + weight of drug into nanoparticles) × 100%

### Analysis of hemocompatibility and immune escape ability of GA-RM/GZ/PL

Different concentrations of GA-RM/GZ/PL and 5% red blood cells were incubated at 37 °C for 2 h and centrifuged at 4000 rpm for 10 min. The absorbance of the supernatant at 545 nm was determined by a multifunctional microplate detector (Tecan, Switzerland). The hemolysis rate was determined according to the absorbance of the supernatant.

Immune escape effect was assessed by the uptake of GA-RM/GZ/PL in macrophages. Macrophages were co-incubated with GZ/PL-Cy5 and GA-RM/GZ/PL-Cy5 (glycyrrhizic acid, 5 μg/ml) for 0.5 h, and then photographed by fluorescence microscopy (Axio, Zeiss, Germany). To quantify the uptake of GA-RM/GZ/PL by macrophages, the cells were lysed with RIPA buffer. After centrifugation at 5000 rpm for 10 min, the supernatant was obtained and used to measure the fluorescence intensity at 670 nm (excitation 650 nm) via a fluorimeter (F4600, Hitachi, Japan).

### *Anti-lipidosis effect of GA-RM/GZ/PL *in vitro

The cellular NAFLD model was incubated with GZ, GZ/PL and GA-RM/GZ/PL (glycyrrhizic acid, 10 μg/ml) for 24 h. The expression of circRNA_0001805, miR-106a-5p, and miR-320a and the mRNA expression of ABCA1 and CPT1 were determined by qRT-PCR. Total TC and TG were detected with commercial kits (Solarbio, Beijing, China). Lipid staining of cultured cells was performed using Oil Red O.

### *Anti-lipidosis effect of GA-RM/GZ/PL *in vivo

To measure the distribution of GA-RM/GZ/PL in the body, mice with NAFLD were injected with GZ/PL-Cy5 and GA-RM/GZ/PL-Cy5 via the tail vein. Mice were euthanized at 6, 24 and 48 h after injection. Then the heart, liver, spleen, lung and kidney were harvested for fluorescence imaging with an In Vivo Imaging System (PerkinElmer, USA). To assess the anti-lipidosis effect, mice with NAFLD were injected with GZ, GZ/PL or GA-RM/GZ/PL (100 μl, glycyrrhizic acid, 2 mg/ml, N = 5) through tail vein once every 2 days, 5 times in total. The weight of the mice was measured every 2 days. On the 20th day after treatment, all the animals were sacrificed, whole blood was collected for hematology, blood biochemical index analysis, and blood calcium detection, and the main organs (tumor, heart, liver, spleen, lungs, and kidneys) were harvested, fixed with 4% paraformaldehyde, embedded in paraffin, and sectioned. Then, the tissue sections were stained with Oil Red O and hematoxylin and eosin and imaged under a microscope (DM2500, Leica, Germany) for histological analysis.

### Data analysis

All experiments in the current study were conducted at least three times and representative results were presented as the means ± SD. Statistically significant differences were compared and evaluated by two-tailed Student’s t-test (between two groups) or by one-way analysis of variance (ANOVA) (among multiple groups) using GraphPad Prism 7.0 (GraphPad, La Jolla, USA). A *P* value smaller than 0.05 was considered significant for all analyses.

## Results

### CircRNA_0001805 expression was downregulated in NAFLD

**Fig. 3:**
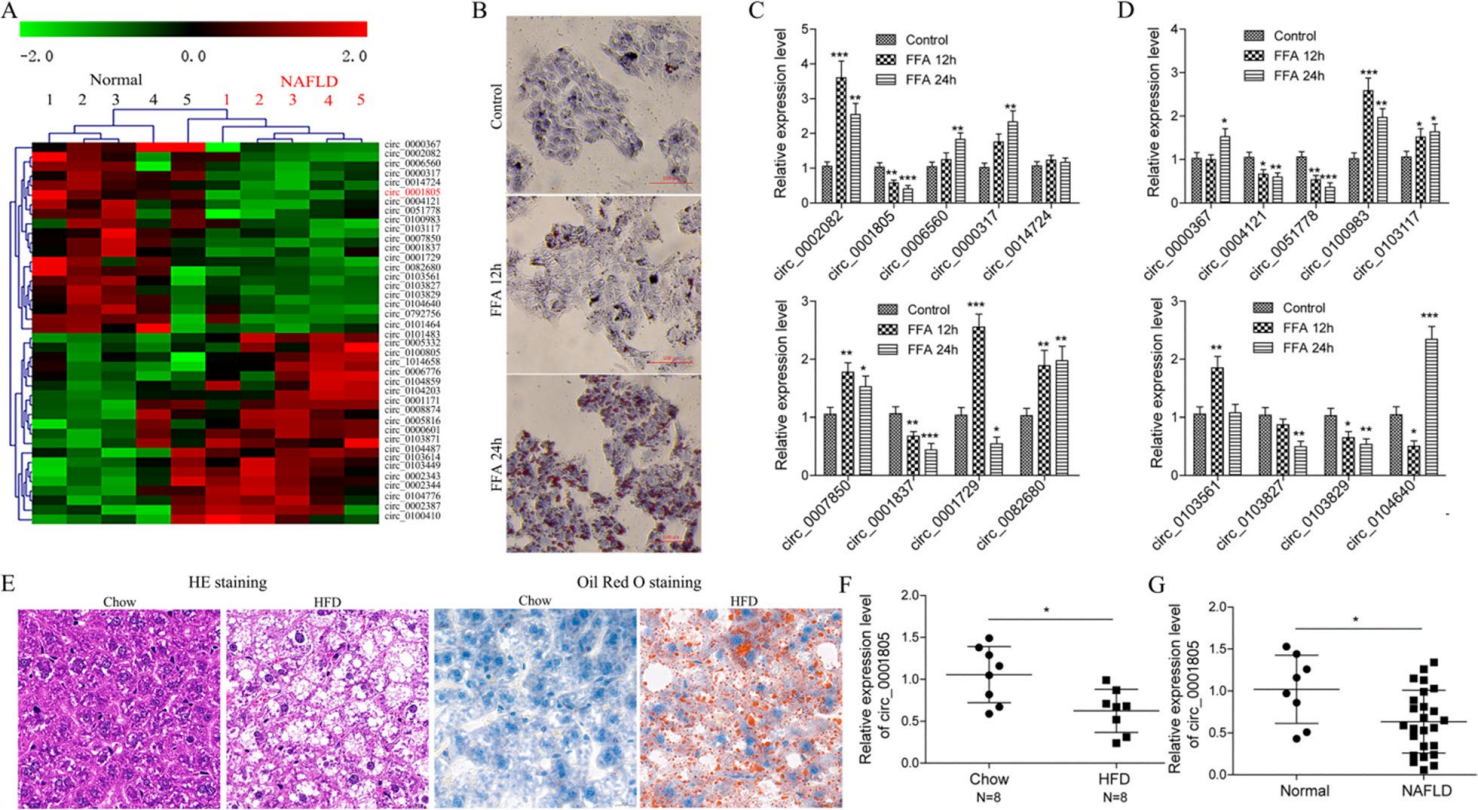
CircRNA_0001805 expression is decreased in NAFLD. **A** The circRNA expression profile in five healthy individuals liver tissues and five NAFLD patients liver tissues by high throughput sequencing. **B** Primary human hepatocytes were incubated with free fatty acids (FFAs), including palmitic acid and oleic acid, for 12 or 24 h. The cells were stained with Oil Red O solution, and lipid droplet accumulation was visualized under a microscope. **C**, **D** Primary human hepatocytes were treated with FFAs for 12 or 24 h, and the relative expression of 18 circRNAs was determined by qRT-PCR. **E** A NAFLD mouse model was established by feeding 8-week-old mice a HFD, and liver samples were examined by H&E or Oil Red O staining. **F** qRT-PCR was performed to detect the expression of the homologous sequence of human circRNA_0001805 in a HFD mouse model. **G** The relative expression of circRNA_0001805 in the liver tissues from NAFLD patients (N = 25) and healthy individuals (N = 9) was compared. The data are shown as the mean ± SD of at least three independent experiments. *P < 0.05, **P < 0.01 and ***P < 0.001

The onset and development of NAFLD are often closely associated with the aberrant expression of hepatic genes, and a recent study by Guo et al. reported that the expression of a total of 357 circRNAs was dysregulated in a hepatic steatosis cellular model; of these circRNAs, 154 were upregulated and 203 were downregulated. In the present study, we first investigated and validated whether the expression profiles of the top eighteen previously identified differentially expressed circular RNAs were altered during NAFLD (Fig. 3A). An in vitro NAFLD model was successfully established by treating primary hepatocytes with FFAs, and gradual accumulation of lipid droplets was observed (Fig. 3B). Consistent with this finding, we observed that the expression of circRNA_0001805, circRNA_0103827, circRNA_0103829, and circRNA_0101837 was significantly downregulated, whereas the expression of circRNA_0006560 was upregulated after 12 or 24 h of FFA treatment. However, our qRT-PCR results revealed that the expression of circRNA_0000317, circRNA_0082680, and circRNA_0103117 was enhanced instead of reduced, whereas the expression of circRNA_0004121 and circRNA_0051778 was reduced instead of enhanced (Fig. 3C, D). Interesestingly, we found that the molecular expression of circRNA_0100983, circRNA_0007850, circRNA_0001729, and circRNA_0103561 first increased (12 h after FFA stimulation) and then decreased (24 h after FFA stimulation), whereas that of circRNA_0002082, circRNA_0104640, and circRNA_0000367 first decreased and then increased.

To validate the observation described above, we established an NAFLD animal model by feeding mice a HFD. Both H&E and Oil Red O staining experiments demonstrated that lipids substantially accumulated in the livers of mice fed a HFD compared with those of mice fed a NCD (Fig. 3E). We next evaluated the expression level of the human circRNA_0001805 homolog in NAFLD mice. qRT-PCR assays on mouse hepatic tissues showed that circRNA_0001805 expression was indeed significantly reduced in mice fed a HFD (N = 8) compared to those fed a NCD (N = 8) (Fig. 3F). Furthermore, as shown in Fig. 3G, we determined the expression profile of circRNA_0001805 in human hepatic tissues and found that circRNA_0001805 expression was markedly lower in NAFLD patients (N = 25) than in normal controls (N = 9). Taken together, our results confirmed the aberrant expression of circRNAs in NAFLD and demonstrated that circRNA_0001805 expression was significantly reduced in FFA-stimulated cells, HFD-fed mice and NAFLD patients.

### In vitro overexpression of circRNA_0001805 attenuated FFA-induced lipid metabolism disorder and hepatic inflammation and modulated the NF-κB pathway and CPT1/ABCA1 expression

**Fig. 4:**
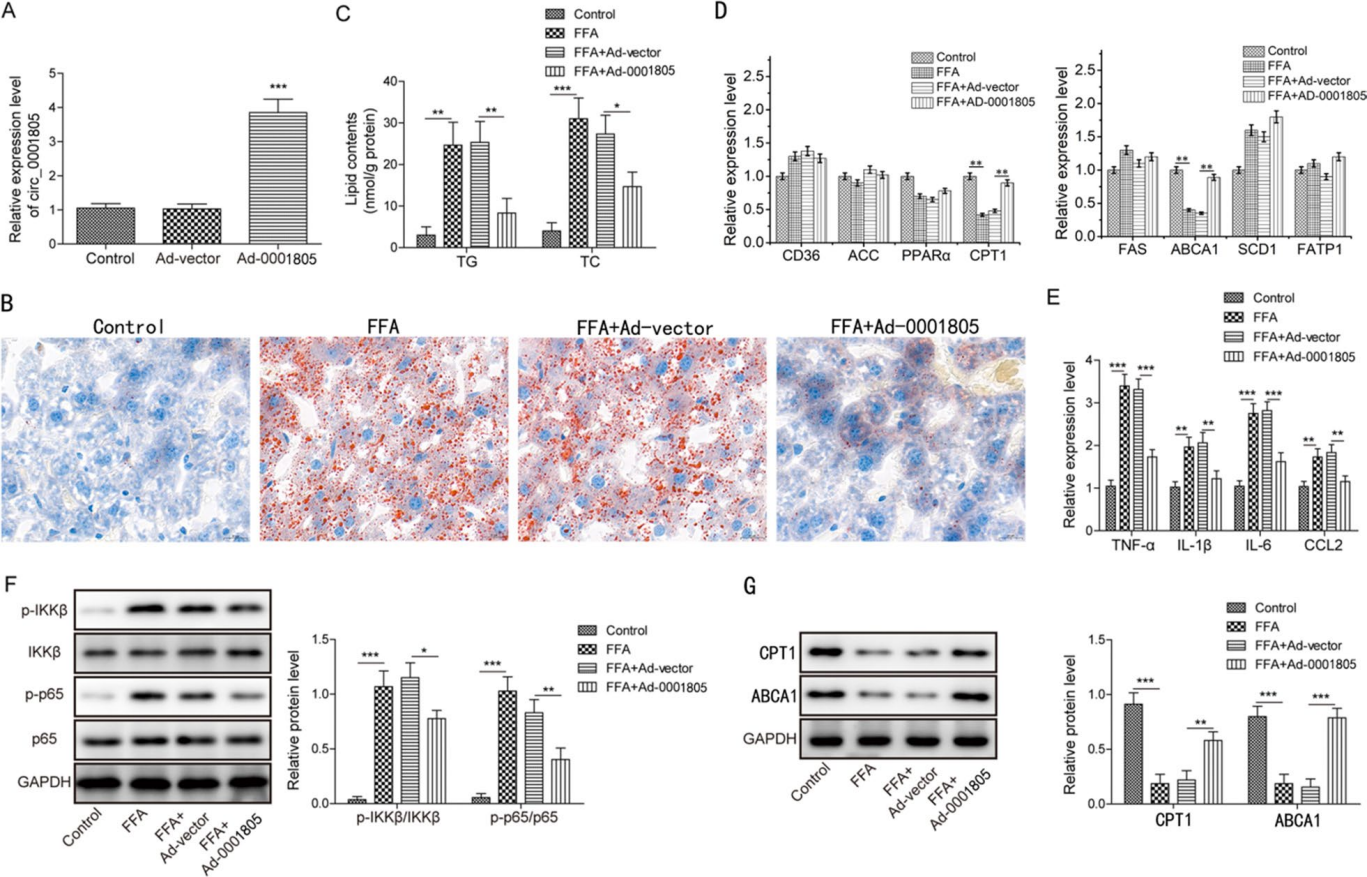
CircRNA_0001805 overexpression ameliorated the NAFLD phenotype in the in vitro model, regulated the expression of lipid metabolism-related genes and attenuated the inflammatory response. **A** Primary hepatocytes were transfected with adenovirus carrying circRNA_0001805 or empty adenovirus, and the expression of circRNA_0001805 was validated by qRT-PCR. **B** Lipid droplet formation was detected by Oil Red O staining. **C** Measurements of total cholesterol (TC) and triglyceride (TG) were conducted, and circRNA_0001805 overexpression decreased both the TC and TG levels, which were induced by FFA treatment. **D** The relative expression of CD36, ACC, PPARα, CPT1, FAS, ABCA1, SCD1 and FATP1 was determined by qRT-PCR. **E** FFA treatment promoted the mRNA expression of proinflammatory cytokines (TNF-α, IL-6, and IL-1β) and the inflammatory chemokine CCL-2, which was inhibited by circRNA_0001805 overexpression. **F** WB analysis was performed to evaluate the expression of NF-κB pathway-related proteins. **G** Protein expression of CPT1 and ABCA1 was measured in circRNA_0001805-overexpressing primary hepatocytes. The data are shown as the mean ± SD of at least three independent experiments. *P < 0.05, **P < 0.01 and ***P < 0.001

To thoroughly explore the role of circRNA_0001805 in the pathology of NAFLD, we overexpressed circRNA_0001805 in primary human hepatocytes using adenoviral vectors (Fig. 4A). Oil Red O staining assays showed that FFA-stimulated lipid accumulation was attenuated in circRNA_0001805-overexpressing primary hepatocytes compared to empty adenovirus-transfected primary hepatocytes (Fig. 4B). Thus, we speculated that circRNA_0001805 might be implicated in lipid metabolism disorder in FFA-treated cells in vitro. As expected, the TC and TG levels, which were enhanced by FFA treatment, were inhibited by circRNA_0001805 overexpression (Fig. 4C). Furthermore, the expression of genes related to lipid metabolism was investigated. The qRT-PCR results indicated that the relative mRNA expression of CPT1 and ABCA1, which was downregulated in FFA-challenged hepatocytes, was significantly diminished by circRNA_0001805 overexpression (Fig. 4D).

Chronic inflammation is another key factor of NAFLD pathogenesis. The expression levels of proinflammatory cytokines (TNF-α, IL-6, and IL-1β) and the inflammatory chemokine CCL-2 are typically enhanced during disease progression. To define the role of the NF-κB pathway in hepatic inflammation, we measured the mRNA levels of TNF-α, IL-6, IL-1β and CCL-2 in primary human hepatocytes treated with FFAs. Interestingly, circRNA_0001805 overexpression effectively inhibited the FFA-induced expression of the inflammatory factors listed above (Fig. 4E). Since the NF-κB pathway is a crucial proinflammatory signaling pathway, we next investigated whether its activation was affected by circRNA_0001805. Notably, FFA stimulation enhanced the levels of phosphorylated IKKβ and p65, the major factors of the NF-κB pathway, whereas the levels of the inactive, unphosphorylated proteins remained unchanged, indicating activation of the NF-κB pathway by FFA treatment (Fig. 4F). In contrast, circRNA_0001805 inhibited the NF-κB cascade. Consistent with this finding, in the current study, we observed that the protein expression levels of CPT1 and ABCA1 were reduced in the FFA-stimulated NAFLD cellular model. Notably, circRNA_0001805 overexpression effectively increased the expression of CPT1 and ABCA1 (Fig. 4G). These findings suggested that circRNA_0001805 might ameliorate NAFLD by inhibiting NF-κB and upregulating CPT1/ABCA1 expression.

### MiR-106a-5p/miR-320a were targets of circRNA_0001805 and directly inhibited ABCA1/CPT1 expression

**Fig. 5:**
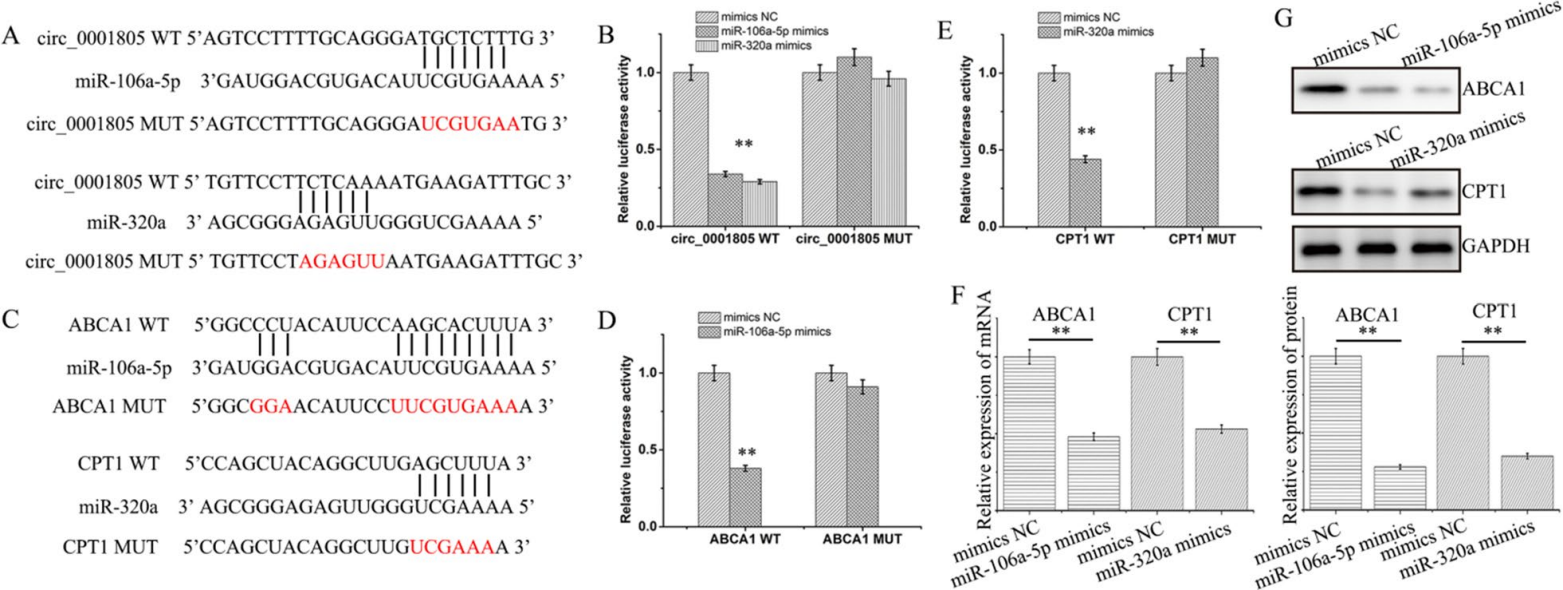
CircRNA_0001805 formed intermolecular interaction with miR-106a-5p and miR-320a, which directly targeted ABCA1 and CPT1. **A** A luciferase reporter assay was designed to study the interactions of circRNA_0001805 with miR-106a-5p and miR-320a. The putative binding sites were predicted using TargetScan, and the mutations in these sites are indicated. **B** The luciferase activity of WT circRNA_0001805 was inhibited by miR-106a-5p or miR-320a overexpression, whereas the luciferase activity of MUT circRNA_0001805 remained unchanged upon cotransfection of the two miRNAs. **C** Potential binding between miR-106a-5p/miR-320a and ABCA1/CPT1 was predicted, and the corresponding in the relevant sequences are indicated. **D** The luciferase activity of WT ABCA1 was decreased upon cotransfection of miR-106a-5p mimic; however, the luciferase activity of MUT ABCA1 remained unchanged. **E** Similar results were observed for CPT1 and CPT1 mutants. Primary hepatocytes were transfected with miR-106a-5p or miR-320a mimics, and the molecular expression of ABCA1 and CPT1 was measured by qRT-PCR (**F**) and WB analysis (**G**). Cells transfected with empty adenoviruses were used as negative controls. The data are shown as the mean ± SD of at least three independent experiments. *P < 0.05, **P < 0.01 and ***P < 0.001

Next, we identified miR-106a-5p and miR-320a as potential target molecules of circRNA_0001805 by using CircInteractome (Fig. 5A). Luciferase assays showed that the relative luciferase activity of WT circRNA_0001805 was significantly reduced when the cells were cotransfected with miR-106a-5p or miR-320a mimics compared with NC mimic. In contrast, the luciferase activity of MUT circRNA_0001805 remained unchanged in the presence of miR-106a-5p, miR-320a or NC mimics (Fig. 5B). Interestingly, our bioinformatics analysis suggested that ABCA1 and CPT1 might be potential targets of miR-106a-5p and miR-320a, respectively (Fig. 5C). We performed a luciferase assay and substantiated the intramolecular interactions between ABCA1 and miR-106a-5p (Fig. 5D) and between CPT1 and miR-320a (Fig. 5E). Moreover, we observed that overexpression of miR-106a-5p and miR-320a significantly reduced the expression levels of ABCA1 and CPT1 at both the mRNA (Fig. 5F) and protein levels (Fig. 5G), respectively. Together, our data demonstrated that miR-106a-5p and miR-320a were direct targets of circRNA_0001805. Furthermore, miR-106a-5p and miR-320a suppressed the molecular expression of ABCA1 and CPT1 through intermolecular interaction.

### In vivo* circRNA_0001805 overexpression rescued HFD-induced lipid metabolism disorder and hepatic inflammation in an NAFLD mouse model*

**Fig. 6:**
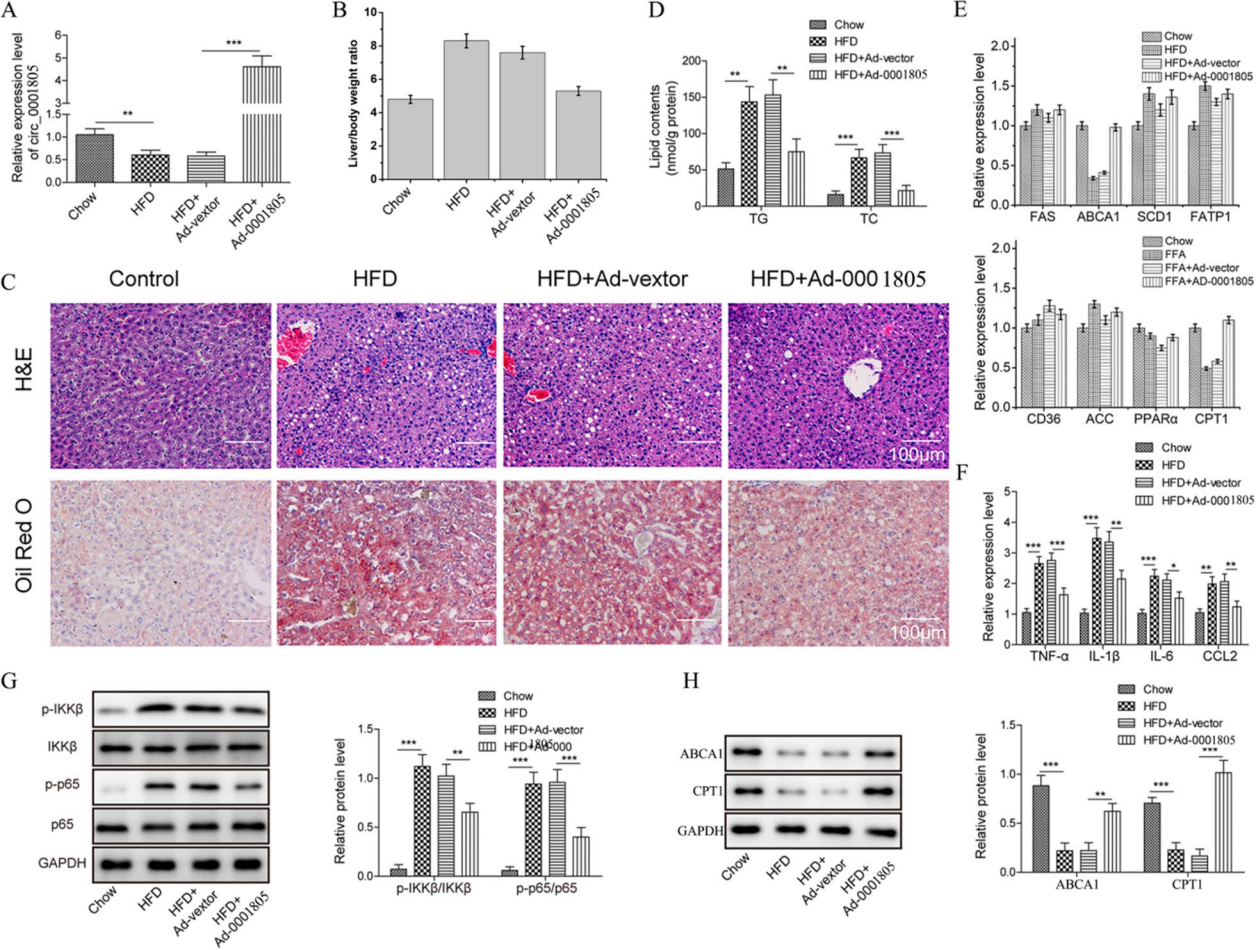
An NAFLD mouse model was established by feeding 8-week-old mice a HFD or normal chow diet as a control. **A** qRT-PCR assays showed that circRNA_0001805 expression in liver tissues was significantly higher in mice injected with circRNA_0001805 adenoviruses than in those injected with empty viruses. **B** CircRNA_0001805 overexpression in mice with NAFLD effectively decreased the liver weight/body weight ratio induced by HFD feeding. **C** Mouse liver sections were prepared in paraffin and examined by H&E staining and Oil Red O staining. Both methods indicated that the HFD-induced hepatic lipid accumulation was effectively reversed by circRNA_0001805 overexpression. **D** The contents of liver lipids (TC and TG) that were enhanced by HFD feeding were largely reduced by circRNA_0001805 overexpression. **E** qRT-PCR was performed to determine the mRNA expression of CD36, ACC, PPARα, CPT1, FAS, ABCA1, SCD1 and FATP1 in mouse livers. **F** The relative expression of proinflammatory cytokines (TNF-α, IL-6, and IL-1β) and the inflammatory chemokine CCL-2 was compared in HFD-treated mice injected with circRNA_0001805 or empty viruses. **G** WB analysis was conducted to evaluate the expression of NF-κB pathway-related proteins. **H** The molecular expression of ABCA1 and CPT1 was examined by WB and qRT-PCR. The expression levels of both molecules, which were inhibited by HFD treatment, were almost completely restored in mice injected with circRNA_0001805 adenoviruses. The data are shown as the mean ± SD of at least three independent experiments. *P < 0.05, **P < 0.01 and ***P < 0.001

To mimic the pathophysiology of human NAFLD, we established an NAFLD animal model by feeding mice a HFD. The animals were injected with circRNA_0001805-carrying adenovirus to achieve stable overexpression (Fig. 6A). Given the close association between diet-induced obesity and NAFLD, we recorded changes in mouse body weight every four days for eight weeks. Compared to the NCD control mice, the HFD-fed mice exhibited a large increase in the liver weight-to-body weight ratio (Fig. 6B), which was markedly reduced in the HFD-fed mice overexpressing circRNA_0001805. H&E and Oil Red O staining showed that lipid droplet formation was significantly reduced in the hepatic tissues of the HFD-fed mice overexpressing circRNA_0001805 compared to those injected with empty adenoviral vectors (Fig. 6C). Additionally, circRNA_0001805 injection largely reduced the HFD-stimulated levels of TG and TC in the hepatic tissues of the mice with NAFLD (Fig. 6D). As expected, we found that the relative mRNA expression of ABCA1 and CPT1, which was downregulated in the HFD-fed mice, was significantly increased in the mice injected with circRNA_0001805 (Fig. 6E). In addition to affecting in vivo NAFLD-induced lipid metabolism disorder, circRNA_0001805 overexpression inhibited HFD-induced hepatic inflammation. qRT-PCR analysis revealed that the expression levels of inflammatory proinflammatory cytokines (TNF-α, IL-6, and IL-1β) and the inflammatory chemokine CCL-2, which were enhanced in the hepatic tissues of mice with NAFLD, were significantly inhibited by circRNA_0001805 overexpression (Fig. 6F). Furthermore, as shown in Fig. 4H and I, circRNA_0001805 overexpression markedly inhibited the HFD-stimulated overactivation of the NF-κB pathway (Fig. 6G) in mice with NAFLD by decreasing the phosphorylation of pathway components. Consistent with our in vitro data, in the hepatic tissues of the HFD-fed mice overexpressing circRNA_0001805, we observed a marked upregulation of ABCA1 and CPT1 expression (Fig. 6H). Collectively, our findings demonstrated that circRNA_0001805 inhibited NF-κB signaling cascades and promoted ABCA1/CPT1 expression in mice; these results corroborated the protective role of circRNA_0001805 against NAFLD-induced lipid metabolism disorder and hepatic inflammation in vivo.

### Protective effect of circRNA_0001805 in NAFLD was essentially mediated by ABCA1 and CPT1

**Fig. 7:**
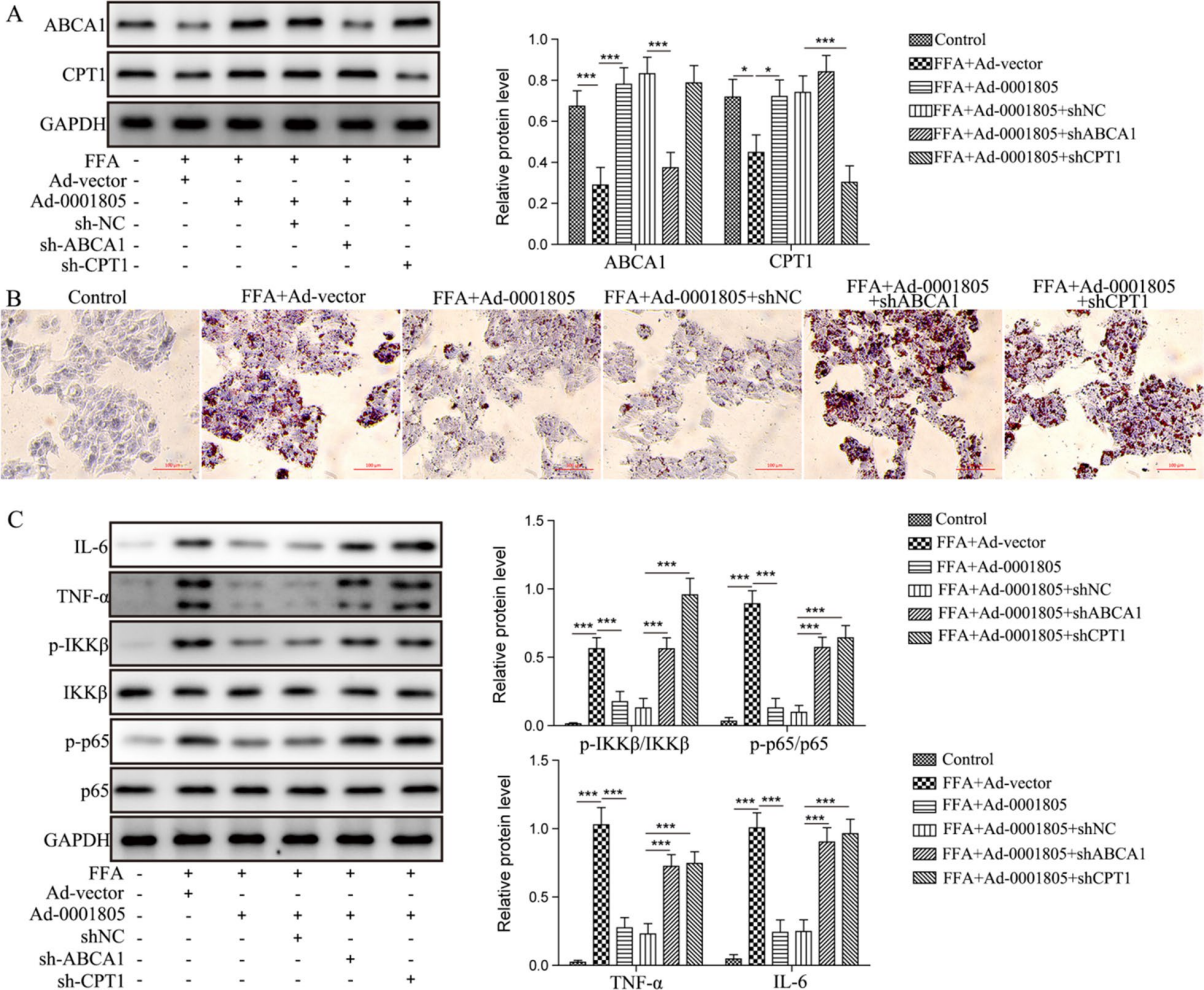
The protective effect of circRNA_0001805 against NAFLD was mediated by ABCA1/CPT1. **A** FFA-treated primary hepatocytes were cotransfected with adenovirus carrying circRNA_0001805- and ABCA1/CPT1-specific shRNAs. The silencing of ABCA1/CPT1 expression with shRNAs was validated by WB analysis. **B** Cells were stained with Oil Red O, and lipid droplet formation was visualized under a microscope. The circRNA_0001805-mediated inhibition of lipid droplet accumulation was greatly suppressed by ABCA1 or CPT1 knockdown. **C** The expression levels of NF-κB pathway-related proteins were assessed by WB analysis. The activity of this pathway, which was attenuated by circRNA_0001805 overexpression, was restored by ABCA1 or CPT1 knockdown. The data are shown as the mean ± SD of at least three independent experiments. *P < 0.05, **P < 0.01 and ***P < 0.001

ABCA1 and CPT1 were previously demonstrated to alleviate atherosclerosis or diabetes by regulating the NF-κB signaling pathway. Our findings raise the question of whether ABCA1 and CPT1 influence the protective effect of circRNA_0001805 against NAFLD. To this end, we transfected circRNA_0001805-overexpressing primary human hepatocytes with shRNAs to achieve the stable knockdown of ABCA1 or CPT1 expression (Fig. 7A). Indeed, the inhibitory effect of circRNA_0001805 on cellular lipid accumulation was partially abolished by silencing ABCA1 or CPT1 expression (Fig. 7B). Moreover, the circRNA_0001805-mediated inhibition of the expression levels of NF-κB pathway components, including IKKβ, p65, TNF-α and IL-6, was largely attenuated by ABCA1 or CPT1 knockdown (Fig. 7C). These findings suggest that ABCA1 and CPT1 are indispensable for the beneficial effects of circRNA_0001805 against lipid metabolism disorder during NAFLD progression, and the molecules exert their effects by closely regulating the NF-κB signaling pathway.

### Protective effect of circRNA_0001805 against NAFLD is closely related to that of miR-106a-5p and miR-320a

**Fig. 8:**
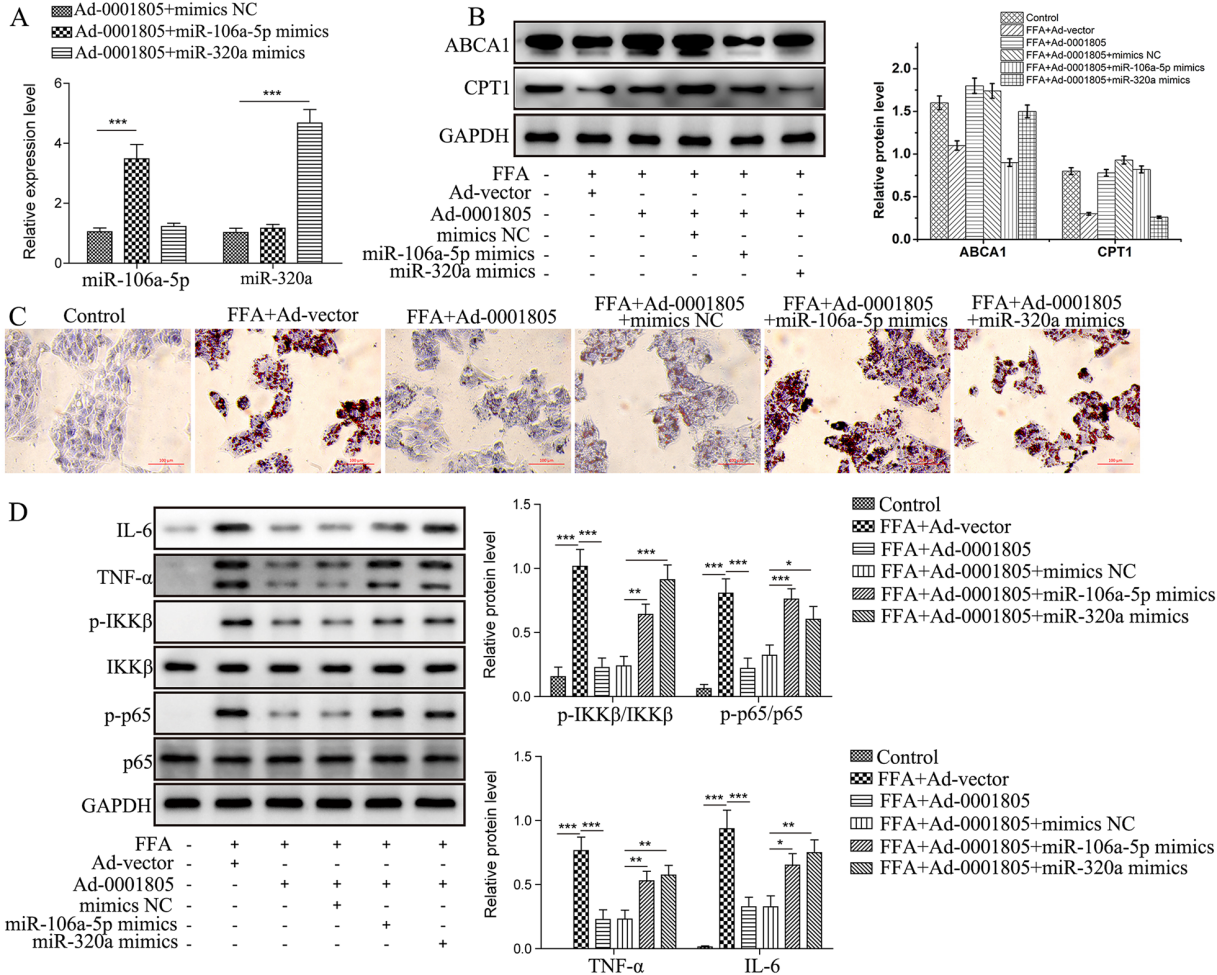
Targeting miR-106a-5p and miR-320a is indispensable for the beneficial role of circRNA_0001805 in NAFLD. **A** Primary hepatocytes were cotransfected with circRNA_0001805 adenovirus and miR-106a-5p, miR-320a or NC mimics, and the relative expression levels of miR-106a-5p and miR-320a were examined by qRT-PCR. **B** FFA-treated primary hepatocytes were cotransfected with adenovirus carrying circRNA_0001805 and miR-106a-5p/miR-320a mimics. The silencing of ABCA1/CPT1 expression by miRNAs was validated by WB analysis. **C** FFA-treated primary hepatocytes cotransfected with circRNA_0001805 and miR-326 or miR-330-5p were stained with Oil Red O, and lipid droplet accumulation was observed under a microscope. **D** The molecular expression of NF-κB pathway-related proteins was determined by WB analysis. The data are shown as the mean ± SD of at least three independent experiments. *P < 0.05, **P < 0.01 and ***P < 0.001

We next addressed the question of whether the protective effect of circRNA_0001805 against NAFLD was affected by miR-106a-5p and miR-320a. Mimics of miR-106a-5p and miR-320a were cotransfected into circRNA_0001805-overexpressing primary human hepatocytes to achieve stable expression (Fig. 8A). In addition, we observed that the effect of circRNA_0001805 in enhancing the expression levels of ABCA1 and CPT1 was partially abolished by miR-106a-5p and miR-320a overexpression (Fig. 8B). Interestingly, the inhibitory effect of circRNA_0001805 on cellular lipid accumulation was attenuated by miR-106a-5p or miR-320a cotransfection (Fig. 8C). Furthermore, our WB results revealed that circRNA_0001805-mediated inhibition of the expression of NF-κB signaling pathway components, including IKKβ, p65, TNF-α and IL-6, was greatly abolished by miR-106a-5p or miR-320a cotransfection (Fig. 8D). These findings suggest that miR-106a-5p or miR-320a plays a pivotal role in rescuing the function of circRNA_0001805 in NAFLD progression by directly modulating the NF-κB signaling pathway.

### Preparation and characterization of GA-RM/GZ/PL

**Fig. 9:**
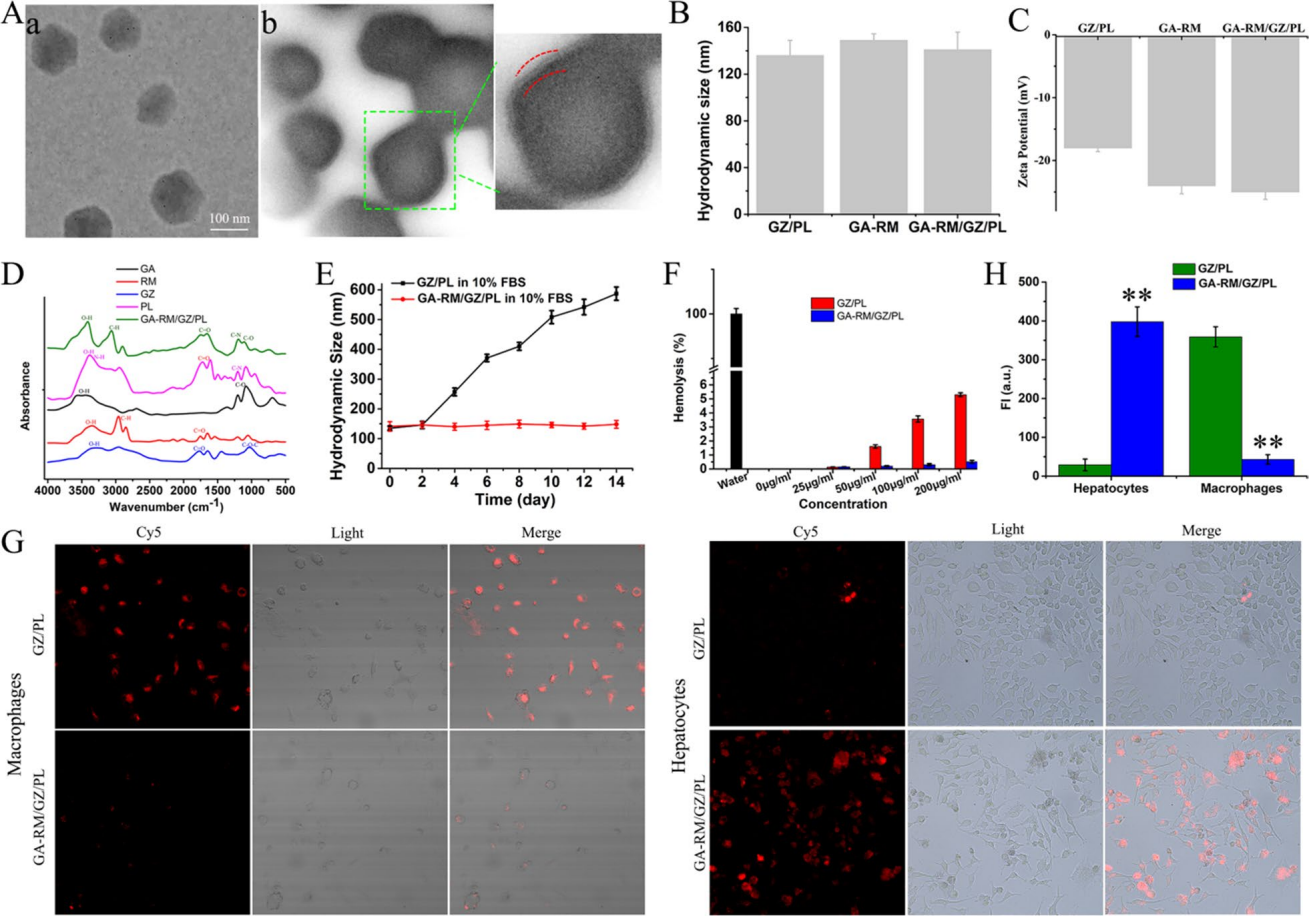
Characterization of GA-RM/GZ/PL. **A** TEM images of GZ/PL (**a**) and GA-RM/GZ/PL (**b**). **B** Hydrodynamics and **C** zeta potentials of GZ/PL, GA-RM and GA-RM/GZ/PL. **D** FTIR spectra of GA, RM, GZ, PL and GA-RM/GZ/PL at 4000–500 cm^−1^. **E** The hydrodynamic sizes of GZ/PL and GA-RM/GZ/PL over 14 days in 10% FBS. **F** Hemolysis of erythrocytes treated with different concentrations of GZ/PL and GA-RM/GZ/PL at 37 °C for 2 h were quantified. **G** Fluorescence images of macrophages and hepatocytes co-incubated with GZ/PL and GA-RM/GZ/PL for 6 h. **H** Fluorescence semi-quantitative analysis of macrophages and hepatocytes co-incubated with GZ/PL and GA-RM/GZ/PL for 6 h. *P < 0.05, **P < 0.01

The process of GA-RM/GZ/PL preparation (Fig. 2) mainly included glycyrrhizic acid and Zn^2+^ to synthesize a metal–organic framework to load circRNA_0001805 plasmid to form a GZ/PL nanocore, galactose-modified RBC membrane vesicles (GA-RM) coated GZ/PL to form GA-RM/GZ/PL. First, as shown in Fig. 9A, TEM image indicated that GA-RM/GZ/PL displayed a core–shell structure. The particle diameter of GZ/PL was about 128 nm. After envelope with RBC membrane vesicles, the RM/GZ/PL particles were 139 nm in size. Furthermore, The dynamic light scattering result (Fig. 9B) indicated that GZ/PL, GA-RM, and GA-RM/GZ/PL particle sizes were 136 nm, 149 nm and 141 nm. As shown in Fig. 9C, the zeta potentials of GZ/PL, GA-RM, and GA-RM/GZ/PL were − 18, − 24 and − 25 mV, respectively. The zeta potential of GA-RM/GZ/PL was close to that of GA-RM. The increase of absolute zeta potential of GZ/PL may be attributed to the charge shielding effect caused by membrane coating, which confirmed the successful construction of GA-RM/GZ/PL. As shown in Additional file 1: Figure S1, agarose gel eletrophoresis indicated that the optimal ratio of GZ to plasmid was 2:1. The encapsulation efficiency (EE) of galactose and plasmid DNA in GA-RM/GZ/PL were 76.5 and 94.3%, and the loading capacity (LC) of galactose and plasmid DNA in GA-RM/GZ/PL were 23.6 and 14.6%. FT-IR assay was used to study the chemical structure and possible interactions of GA-RM/GZ/PL. The FT-IR spectra of GA, RM, GZ, PL and GA-RM/GZ/PL were indicated in Fig. 9D. The peak of GA at 3350 cm^−1^ corresponds to the O–H group, C-O bend exists at 1170 and 1140 cm^−1^ [33]. Due to the presence of lipids, proteins, carbohydrates and other molecules, the FTIR spectra of RM was complex [34]. The stretching vibrations of the lipid hydrocarbon locates at the range of 2950–2850 cm^−1^. The C=O absorption exists around 1800 cm^−1^. Moreover, the absorption peak of phosphates and carbohydrates mainly locates at the range of 1250–1000 cm^−1^. The characteristic peaks of GZ were 3350–3200 cm^−1^ (O–H), 1740 cm^−1^(C=O), and 1055 cm^−1^(C–O–C) [35]. As for plasmid DNA [36], the peak between 3700 and 3000 cm^−1^ represents O–H and N–H stretching vibrations, the peaks at 1690, 1646, 1712 and 1486 cm^−1^ represent adenine, thymine, guanine and cytosine, respectively. GA-RM/GZ/PL had the characteristic peak of GA, RM, GZ and PL at 3400 cm^−1^ (O–H), 3050 cm^−1^ (C–H), 1750 cm^−1^ (C=O), 1200 cm^−1^ (C–N), 1100 cm^−1^ (C–O). In order to detect the dispersion and stability of GA-RM/GZ/PL, the particle sizes of GZ/PL and GA-RM/GZ/PL were evaluated over two weeks (Fig. 9E). The hydrodynamic size of GZ/PL in 10% fetal bovine serum (FBS) obviously enlarged with time, while there was no obvious change in particle size of GA-RM/GZ/PL. After envelope of RBC membrane, GA-RM/GZ/PL possessed good stability in physiological solution.

Next, the hemocompatibility and immune escape ability of GA-RM/GZ/PL were detected. Different concentrations of GZ/PL and GA-RM/GZ/PL were co-incubated with 5% RBCs for 2 h, then the absorbance of the supernatants was determined after centrifugation. In Fig. 9F, the hemolysis rate with GZ/PL (200 µg/ml) was 5.3%, while GA-RM/GZ/PL did not induce significant hemolysis (< 1%). The results indicated that GA-RM/GZ/PL has good blood compatibility and can be used for blood circulation in vivo. The immune escape function of GA-RM/GZ/PL-Cy5 was explored by evaluating the antiphagocytic effect on macrophages. As shown in Fig. 9G, H, in GZ/PL-Cy5-treated group, a large amount of Cy5 (red fluorescence) gathered in the macrophages. While in GA-RM/GZ/PL-Cy5-treated group, the red fluorescence in the macrophages was significantly decreased, indicating that the phagocytosis of GA-RM/GZ/PL-Cy5 by macrophages was obviously decreased after RBC membrane camouflage. The above results confirmed that GA-RM/GZ/PL can reduce the recognition by macrophages and clearance of the immune system. Finally, the targeting ability of GA-RM/GZ/PL was tested. As shown in Fig. 9G, H, compared with GZ/PL-Cy5 group, a large number of Cy5 fluorescence appeared in hepatocytes in GA-RM/GZ/PL-Cy5 group, which confirmed that a large number of GA-RM/GZ/PL-Cy5 was endocytosed into hepatocytes under the action of GA targeting.

### *Anti-lipidosis effect of GA-RM/GZ/PL *in vitro

**Fig. 10:**
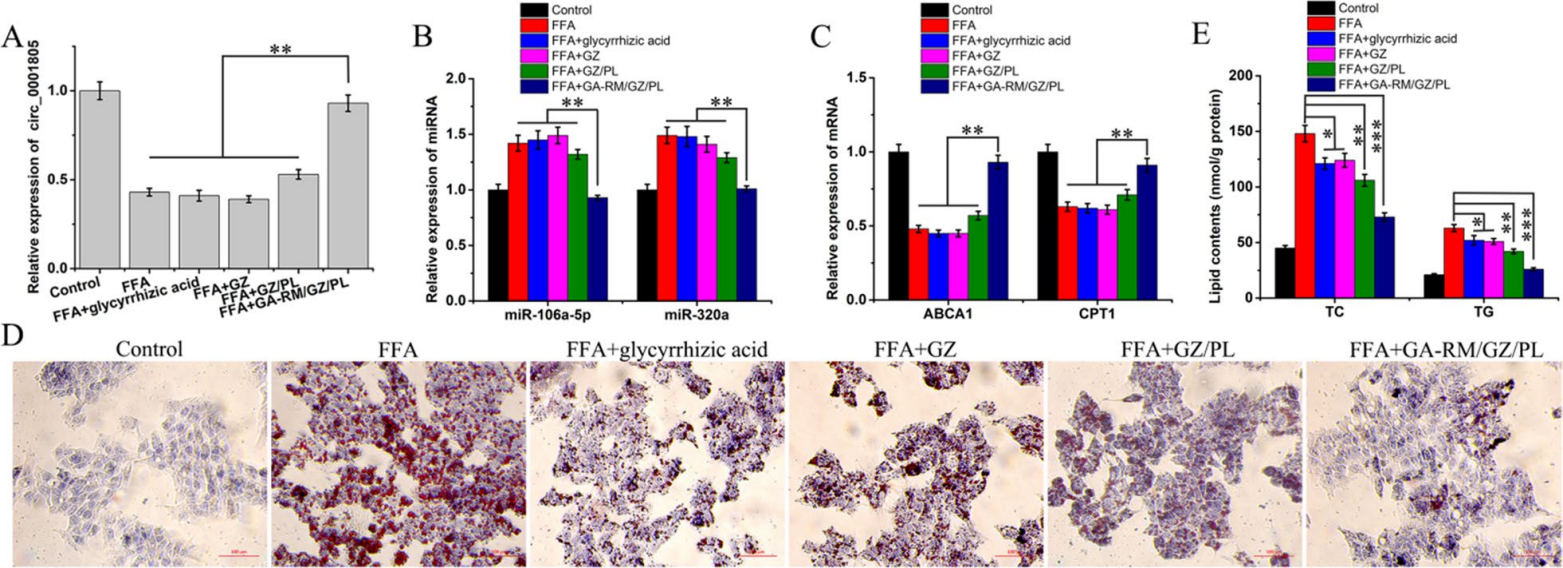
The expression of circRNA_0001805 (**A**), miR-106a-5p, miR-320a (**B**), ABCA1 and CPT1 (**C**) was validated by qRT-PCR. **D** Lipid droplet formation in hepatocytes was determined by the Oil Red O staining. **E** Total cholesterol (TC) and triglyceride (TG) levels were measured, and GA-RM/GZ/PL decreased both the TC and TG levels, which were induced by FFA treatment. *P < 0.05, **P < 0.01

As shown in Additional file 1: Figure S2, compared with adenovirus, there was no signaficant difference in transfection efficiency of GA-RM/GZ/PL. In addition, GA-RM/GZ/PL also has the advantages of security and economy. To thoroughly explore the role of circRNA_0001805 in NAFLD pathology, we overexpressed circRNA_0001805 in primary human hepatocytes using GA-RM/GZ/PL (Fig. 10A). Moreover, in FFA-treated hepatocytes, compared with glycyrrhizic acid, GZ and GZ/PL, GA-RM/GZ/PL significantly decreased miR-106a-5p and miR-320a expression (Fig. 10B) and increased ABCA1 and CPT1 expression (Fig. 10C). As shown in Fig. 10D, the Oil Red O staining showed that glycyrrhizic acid, GZ, GZ/PL and GA-RM/GZ/PL all reduced FFA-stimulated lipid accumulation in primary hepatocytes to some extent. There was no statistical difference of lipid accumulation between glycyrrhizic acid and GZ treatments, which was due to the protective effects of glycyrrhizic acid released from GZ in NAFLD. Notably, compared with glycyrrhizic acid, GZ and GZ/PL, GA-RM/GZ/PL has a more significant effect on attenuating lipid accumulation in primary hepatocytes, which was due to the higher stability of GA-RM/GZ/PL after the inclusion of GA-RM as well as to the specific recognition of GA-RM/GZ/PL by the mannose receptor in the liver cell membrane. After GA-RM/GZ/PL was internalized into hepatocytes, glycyrrhizic acid was released and circRNA_0001805 was highly expressed, which exerted synergistic anti-lipid aggregation effect in NAFLD. Consistent with the results of Oil Red O staining, the TC and TG levels, which were enhanced by FFA treatment, were conversely inhibited by GA-RM/GZ/PL (Fig. 10E).

**Fig. 11:**
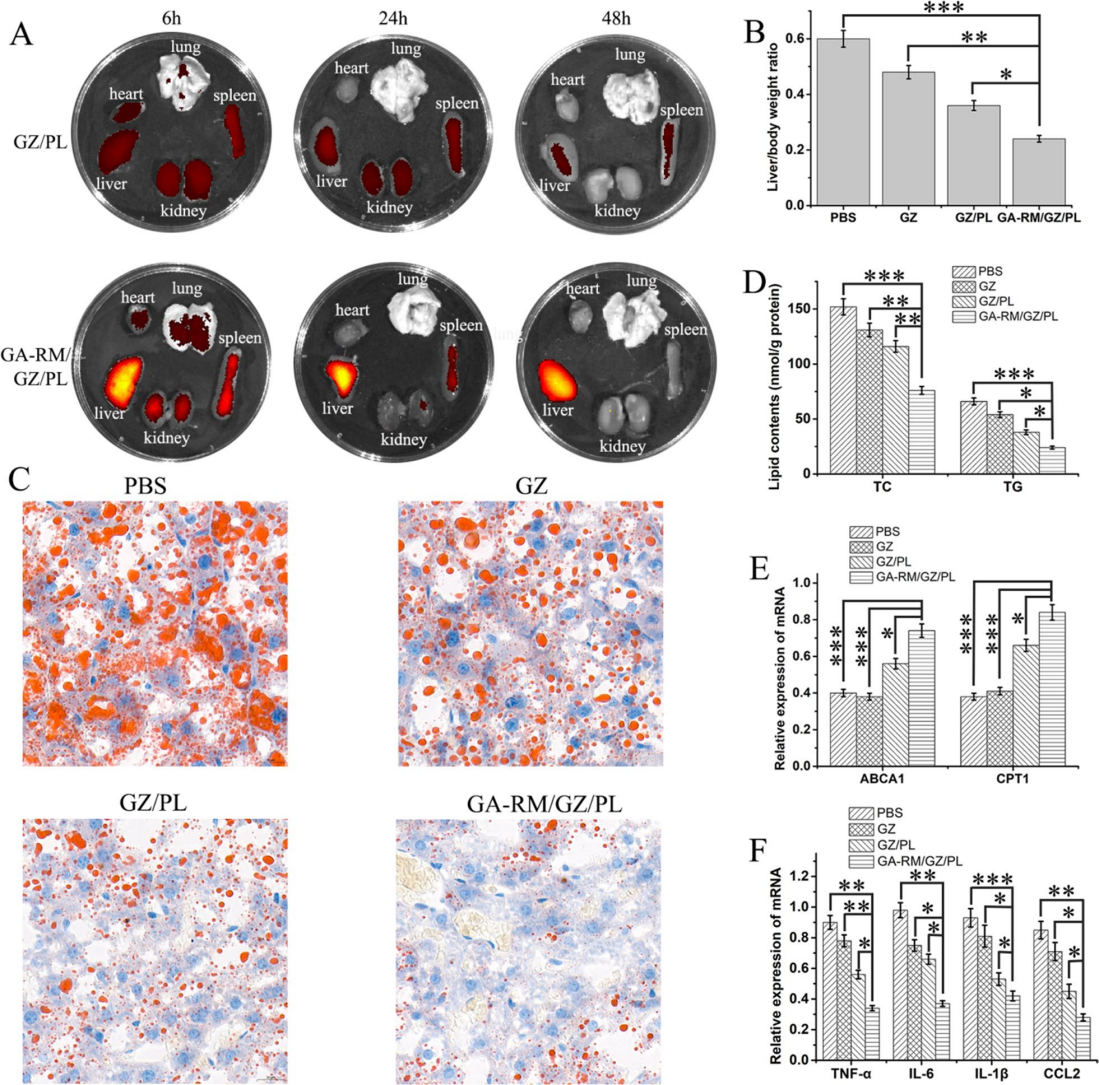
**A** Fluorescence images of the main organs upon treatment with GZ/PL and GA-RM/GZ/PL for 6 h, 24 h and 48 h. **B** In mice with NAFLD, GA-RM/GZ/PL effectively decreased the liver weight/body weight ratio that was induced by an HFD. **C** Mouse liver sections were prepared in paraffin and examined by Oil Red O staining. **D** The contents of liver lipids (TC and TG), which were enhanced by HFD feeding, were largely reduced by GA-RM/GZ/PL. **E** The mRNA expression of ABCA1 and CPT1 was determined by qRT-PCR. **F** The relative expression of proinflammatory cytokines (TNF-α, IL-6, and IL-1β) and the inflammatory chemokine CCL-2 was determined by qRT-PCR in mouse livers. *P < 0.05, **P < 0.01 and ***P < 0.001

**Fig. 12:**
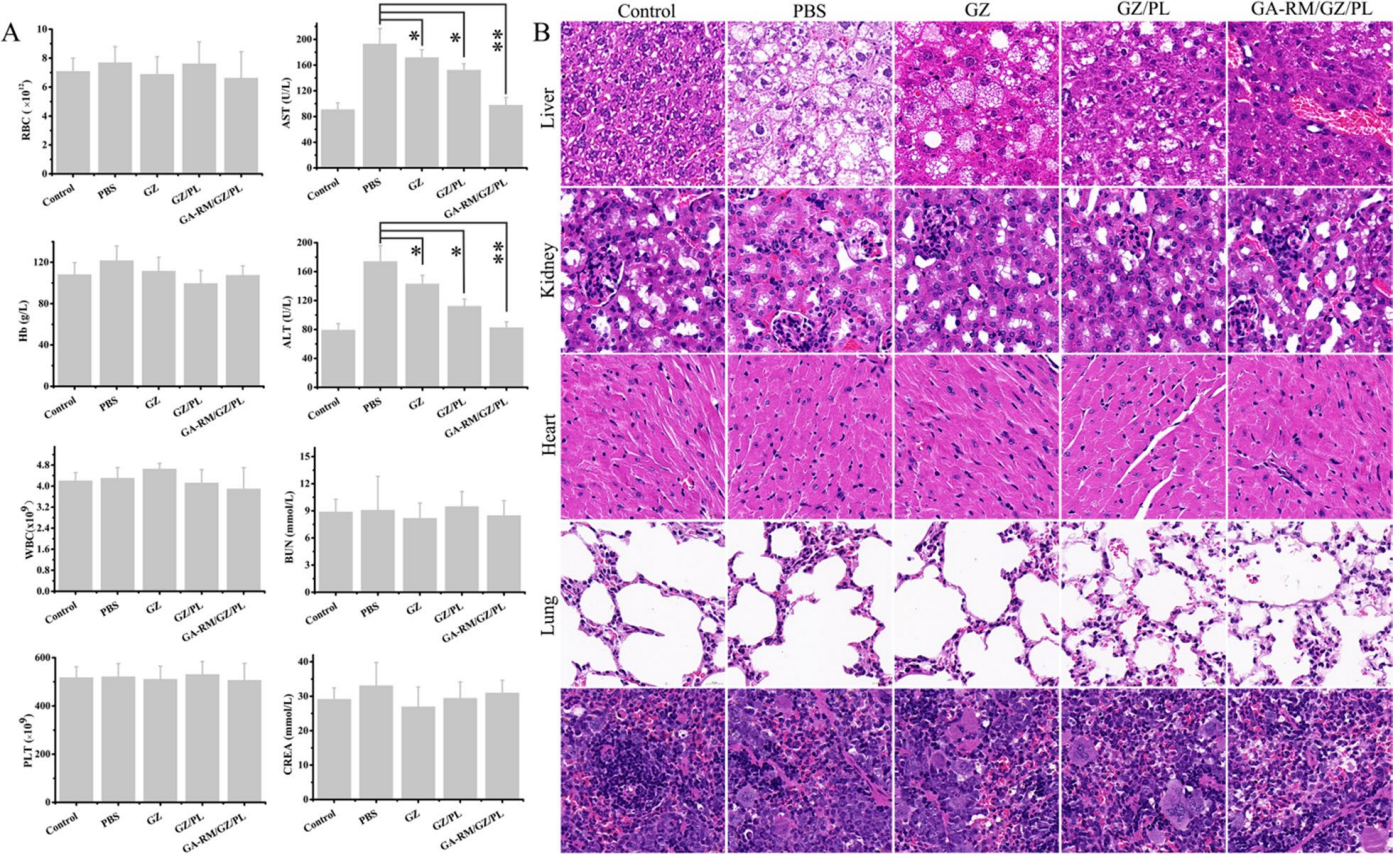
**A** Changes in hematological and biochemical indexes in each group (N = 5). **B** Representative HE staining images of liver, kidney, heart, lung and spleen collected at 20 days after different treatments. *P < 0.05, **P < 0.01 and ***P < 0.001

### *Anti-lipidosis effect of GA-RM/GZ/PL *in vivo

To mimic the human pathophysiology of NAFLD, we established an NAFLD animal model by feeding mice a HFD. First, the biodistribution of GA-RM/GZ/PL in vivo was explored. GZ/PL and GA-RM/GZ/PL were administered through the tail vein. In vivo animal imaging was used to detect the biological distribution of nano-drugs in NAFLD mice for 6–48 h after injection. As shown in Fig. 11A, GZ/PL was observed at low concentrations in the liver from 6 to 48 h. However, under the effect of immune evasion and galactose targeting, the aggregation of GA-RM/GZ/PL at the fatty liver was obviously increased from 24 to 48 h. Second, the antilipidosis effect of GA-RM/GZ/PL in vivo was explored. Mice with NAFLD were injected with PBS, GZ, GZ/PL or GA-RM/GZ/PL via the tail vein. As shown in Fig. 11B, the HFD-fed mice exhibited a large increase in the liver weight-to-body weight ratio, which was markedly reduced in the HFD-fed mice treated with GA-RM/GZ/PL. Oil Red O staining showed that lipid droplet formation was significantly reduced in the hepatic tissues of the HFD-fed mice treated with GA-RM/GZ/PL compared to those injected with GZ and GZ/PL (Fig. 11C). Additionally, GA-RM/GZ/PL injection substantially reduced the HFD-stimulated expression levels of TG and TC in the hepatic tissues of the mice with NAFLD (Fig. 11D). As expected, we found that the relative mRNA expression of ABCA1 and CPT1, which was downregulated in the HFD-fed mice, was significantly increased in the mice injected with GA-RM/GZ/PL (Fig. 11E). In addition to affecting NAFLD-induced lipid metabolism disorder in vivo, GA-RM/GZ/PL also inhibited HFD-induced hepatic inflammation. qRT-PCR analysis revealed that the expression levels of proinflammatory cytokines (TNF-α, IL-6, and IL-1β) and the inflammatory chemokine CCL-2 were enhanced in the hepatic tissues of mice with NAFLD and were significantly inhibited by GA-RM/GZ/PL (Fig. 11F).

Compared with NCD-fed mice, severe impairment of liver function is manifested by elevated AST and ALT levels (Fig. 12A) and hepatocyte steatosis (Fig. 12B) in HFD-fed mice. GZ, GZ/PL and GA-RM/GZ/PL alleviated hepatocyte lipid accumulation and liver function impairment to some extent, and GA-RM/GZ/PL had the most significant therapeutic effect. Furthermore, GA-RM/GZ/PL significantly reduced the values of ALT and AST, and improved liver function. However, erythrocyte, hemoglobin, white blood cell, platelets and renal function index (CREA, BUN) had no significant change (Fig. 12A). Moreover, the results of H&E staining showed that GA-RM/GZ/PL treatment significantly improved hepatocyte steatosis and reduced lipid droplets in the liver, and did not cause histological change in heart, spleen, lung and kidney tissues (Fig. 12B). These results further verify that GA-RM/GZ/PL possesses good biocompatibility and a obvious protective effect against NAFLD.

GA-RM/GZ/PL has good liver targeting ability. By substantially upregulating the expression of circRNA_0001805, ABCA1 and CPT1, GA-RM/GZ/PL can release glycyrrhizic acid to reduce the inflammatory response and play a synergistic role to protect against NAFLD-induced lipid metabolism disorder.

## Discussion

The present study investigated the molecular expression of and interactions among circRNA_0001805, miR-106a-5p/miR-320a and ABCA1/CPT1 during NAFLD development. We demonstrated that circRNA_0001805 expression was decreased in NAFLD cellular and animal models as well as in hepatic tissues obtained from NAFLD patients. Notably, circRNA_0001805 overexpression effectively alleviated NAFLD-induced lipid droplet accumulation, lipid metabolism disorder and inflammation in both FFA-treated primary human hepatocytes and HFD-fed mice. Our results supported the notion that circRNA_0001805 exerts a protective effect against NAFLD by interacting with miR-106a-5p/miR-320a, thus restoring the decreased expression of ABCA1/CPT1 and inhibiting NF-κB signaling.

CircRNAs, a class of long noncoding RNAs, have gained increasing attention in recent years due to their close association with the pathogenesis of NAFLD [37, 38]. However, our understanding of their exact roles and the underlying molecular mechanisms in NAFLD is still in its infancy and remains incomplete. In the present study, we revealed that circRNA_0001805 alleviated NAFLD through a novel competing endogenous RNA (ceRNA) mechanism. These findings increase our knowledge about the regulatory actions of circRNAs in NAFLD. Furthermore, by applying bioinformatics tools and conducting cellular assays, we demonstrated that circRNA_0001805 competitively targeted miR-106a-5p/miR-320a, rather than a single target, and led to much more evident phenotypic changes by simultaneously altering the levels of both miRNA targets, namely, ABCA1 and CPT1. Our research sheds light on the circRNA-mediated fine-tuning of gene expression during NAFLD development, and we anticipate that comprehensive characterization of the downstream miRNA “targetome” network may help us better elucidate the cellular functions of ceRNAs in diverse human diseases, including NAFLD. Thus, our future research will further focus on the target miRNAs that were identified in the present study and explore their collaborative roles in NAFLD.

NAFLD is recognized as a multisystem pathological disease, and its clinical development is closely associated with metabolic syndromes, including hypertension, hyperlipidemia and diabetes mellitus [39]. Although early-stage NAFLD hardly causes immediate damage, accumulating evidence has shown that a severe form of NAFLD accompanied by hepatic and extrahepatic complications is highly related to elevated hepatic inflammatory responses [40]. On the other hand, the increased uptake of free fatty acids and triglycerides from circulation and their dysregulated hepatic accumulation play a central role in the progression of NAFLD to NASH [41, 42]. In the present study, we revealed that circRNA_0001805 upregulation effectively decreased the inflammation levels induced by FFA or HFD treatment and repressed the NF-κB proinflammatory pathway, suggesting that the anti-inflammatory effect of circRNA_0001805 might be exerted through the NF-κB pathway. Moreover, we demonstrated that circRNA_0001805 alleviated NAFLD-induced lipid metabolism disorder and reduced lipid accumulation both in vitro and in vivo. Future research will aim to investigate whether circRNA_0001805 also mitigates insulin resistance and fibrosis and to explore the underlying mechanisms.

With the development of nanotechnology, a large number of nanomaterials have been used as drug carriers in the biomedical field [43, 44]. However, the entry of traditional nanomaterials into the body as exogenous substances leads to immune clearance and affects their bioavailability [45]. Moreover, some nanomaterials have the disadvantages of poor dispersion, poor stability and low drug loading rates [46]. Currently, researchers are beginning to focus on materials from natural sources, such as cell membranes. Nanoparticles camouflaged by cell membrane have the characteristics of both source cell membrane and nano-drug nucleus. Currently, the membranes used to package nanoparticles come from red blood cells, white blood cells, platelets, macrophages, T lymphocytes, stem cells, and tumor cells [47–50]. Nanoparticles have different functions during drug delivery due to different cell membrane sources. In this study, a novel metalorganic framework nanocarrier (GZ) was successfully prepared from glycyrrhizic acid (GA) and zinc ions (Zn^2+^), and this nanocarrier was loaded with a circRNA_0001805 plasmid to construct a nanocore (GZ/PL). Finally, a nanomedicine system (GA-RM/GZ/PL) was prepared by coating GZ/PL with a galactose-modified RBC membrane (GA-RM). The natural erythrocyte membrane endowed GA-RM/GZ/PL with immune evasion properties and long circulation times. This effect is mainly attributed to the fact that CD47 on the erythrocyte membrane was recognized as a "do not eat me" signal by siRPα expressed on phagocytes, thus inhibiting the phagocytosis of the immune cells on the erythrocytes. Glycyrrhizic acid and the circRNA_0001805 plasmid were released after GA-RM/GZ/PL reached liver cells. circRNA_0001805 regulated the NF-κB signaling pathway through miR-106a-5p/miR-320a and ABCA1/CPT1 to reduce the inflammatory response and promote lipid metabolism. Glycyrrhizic acid is the most important active ingredient in licorice. Glycyrrhizic acid itself inhibits NAFLD lipid deposition and exerts anti-inflammatory and detoxification effects.

## Conclusion

In summary, the present study revealed the protective role of circRNA_0001805 against NAFLD-induced fatty acid metabolism disorder and inflammation through the direct regulation of the miR-106a-5p/miR-320a/ABCA1/CPT1 axis. GA-RM/GZ/PL played a synergistic role against NAFLD-induced lipid metabolism disorder. By highlighting the potential value of circRNA_0001805 as a promising drug target for NAFLD treatment, our work provides deeper insight into ceRNA-mediated NAFLD pathogenesis and provides a new nanotherapy strategy for the treatment of NAFLD.

## Supplementary Information

**Additional file 1: Table S1**. Primers and RNA sequences used in this study. **Figure S1**. (A) The optimal ratio of GZ to plasmid was determined by agarose gel eletrophoresis. (B) The encapsulation efficiency and loading capacity of galactose and plasmid DNA in GA-RM/GZ/PL. **Figure S2**. The comparation of transfection efficiency in GA-RM/GZ/PL and adenovirus treated hepatocytes.

## Data Availability

All data generated or analyzed during this study are included in this published article.

## Abbreviations

NAFLD: Non-alcoholic fatty liver disease
NASH: Non-alcoholic steatohepatitis
HCC: Hepatocellular carcinoma
T2DM: Type 2 diabetes mellitus
circRNAs: Circular RNAs
RBPs: RNA-binding proteins
DMEM: Eagle’s Minimal Essential Medium
FBS: Fetal bovine serum
FFA: Free fatty acids
HFD: High fat diet
NCD: Normal chow diet
H&E staining: Hemotoxylin and Eosin staining
WB: Western blot analysis
SDS-PAGE: Sodium dodecyl sulfate polyacrylamide gel electrophoresis
qRT-PCR: Quantitative RT-PCR
SD: Standard deviation
ANOVA: Analysis of variance
