# Supplementary material for: A nanodrug system overexpressed circRNA_0001805 alleviates nonalcoholic fatty liver disease via miR-106a-5p/miR-320a and ABCA1/CPT1 axis
Source: J Nanobiotechnology. 2021 Nov 17;19:363. doi: 10.1186/s12951-021-01108-8 (PMC8596892; doi:10.1186/s12951-021-01108-8)
Supplement: Supplementary file 1 — Additional file 1: Table S1. Primers and RNA sequences used in this study. Figure S1. (A) The optimal ratio of GZ to plasmid was determined by agarose gel eletrophoresis. (B) The encapsulation efficiency and loading capacity of galactose and plasmid DNA in GA-RM/GZ/PL. Figure S2. The comparation of transfection efficiency in GA-RM/GZ/PL and adenovirus treated hepatocytes. [file 12951_2021_1108_MOESM1_ESM.docx]

**Table S1 Primers and RNA sequences used in this study.**

| **Primer sequence** |  |  |
| --- | --- | --- |
| circ_0001805 | Forward | TTTTGAAGATAAACCGAAACCTTC |
|  | Reverse | ACATCCCCATCATGTTCCAT |
| circ_0002082 | Forward | AAAGGCTGAGTGTTGAGGAAA |
|  | Reverse | GTTCTTCCGCTCAAATCCTG |
| circ_0006560 | Forward | CTTGCAGTTGAAATGGATGC |
|  | Reverse | AGAGCTGTACCATCTCCAAGAG |
| circ_0000317 | Forward | GTGATCTGAAAGGGCCAGAG |
|  | Reverse | TCCACATCACCCTTCACCTT |
| circ_0014724 | Forward | GCCAAGTCCATGATCGAAAT |
|  | Reverse | CTGCAATAGTCTTGGCAGCA |
| circ_0000367 | Forward | ATCGAAGACTGGCGTGAAAC |
|  | Reverse | ATTGCACAGTGGATGGATCA |
| circ_0004121 | Forward | TTGCTGGGAACATCAACAGA |
|  | Reverse | TCAGACAAAGGGGATCCAAC |
| circ_0051778 | Forward | GGTGCTGCCGTTGATTTTT |
|  | Reverse | GGCAGAGAATGACGAACAGG |
| circ_0100983 | Forward | AGGTGTAGTTCTGCGAGCAT |
|  | Reverse | AAAATCAATCAAAAACACGTCA |
| circ_0103117 | Forward | GCTGGCATGGAACAAAGTTA |
|  | Reverse | TGCAGAACCTTGAGCTGAGA |
| circ_0007850 | Forward | TCCCAATGCAGAAAAGAAGG |
|  | Reverse | TCGTACTGTATGTGCAGGATCTC |
| circ_0001837 | Forward | ACACGGACACGGATCTTCAT |
|  | Reverse | ATGCTCCTTGCAGAATCCAC |
| circ_0001729 | Forward | AGGCCCTAGCAGCAACAAG |
|  | Reverse | ACTCCGCTGCTCCATCATTA |
| circ_0082680 | Forward | TGAGGCAATTGACATGTGGT |
|  | Reverse | GGCACTTGATTGAAGGGTGT |
| circ_0103561 | Forward | CCAGGGTTATAGCCAGGACA |
|  | Reverse | AGGGATTCTCCGAGTCAGGT |
| circ_0103827 | Forward | TGCATATGTGGCACCTTCAT |
|  | Reverse | GGACTTCCTTCTCTTTCTCCATC |
| circ_0103829 | Forward | TCTGCATATGTGGCACCTTC |
|  | Reverse | TCTCCTTTTTACTTCATGATGATCC |
| circ_0104640 | Forward | TTGGGAGACTCTGTCACAACA |
|  | Reverse | GGGTGCTGTATTTCCCACAC |
| GAPDH | Forward | AATGGGCAGCCGTTAGGAAA |
|  | Reverse | GCGCCCAATACGACCAAATC |
| CPT1 | Forward | AGATCCCAGCCACTGAGCTA |
|  | Reverse | CCAGCCGAGCATCCTATGAG |
| ABCA1 | Forward | TGTCCAACTAAACCGGAGGC |
|  | Reverse | ATGCAGACCTCTTCGGGTTG |
| CD36 | Forward | AAGTTCCTGTCCATCGTGTCC |
|  | Reverse | TCCCGTCACTCTTCCCTCTT |
| ACC | Forward | TGTCAAGACCCGGGTGAGTA |
|  | Reverse | GAACCACACCGTGTTGGGAA |
| PPARα | Forward | CCTGTCTGCTCTGTGGACTC |
|  | Reverse | TGAAAGCGTGTCCGTGATGA |
| FAS | Forward | ACTGTGACCCTTGCACCAAA |
|  | Reverse | AGACAAAGCCACCCCAAGTT |
| SCD1 | Forward | CTTGCGATATGCTGTGGTGC |
|  | Reverse | CCGGGGGCTAATGTTCTTGT |
| FATP1 | Forward | CTCTCTGCTTCCCCAGGATG |
|  | Reverse | GTTTCCTGCCGAGTGGTACA |
| miR-106a-5p | Forward | AATTGTGTTGGCCCAGCTCT |
|  | Reverse | CAGCCTGCCCAGGTCAATAA |
| miR-320a | Forward | CCAGAACTAGGACTGGGTGC |
|  | Reverse | CAGCAGGCACTTGCTCTGTA |
| TNF-α | Forward | CTGGGCAGGTCTACTTTGGG |
|  | Reverse | CTGGAGGCCCCAGTTTGAAT |
| IL-6 | Forward | GTCCAGTTGCCTTCTCCCTGG |
|  | Reverse | CCCATGCTACATTTGCCGAAG |
| CCL2 | Forward | GATCTCAGTGCAGAGGCTCG |
|  | Reverse | TTTGCTTGTCCAGGTGGTCC |


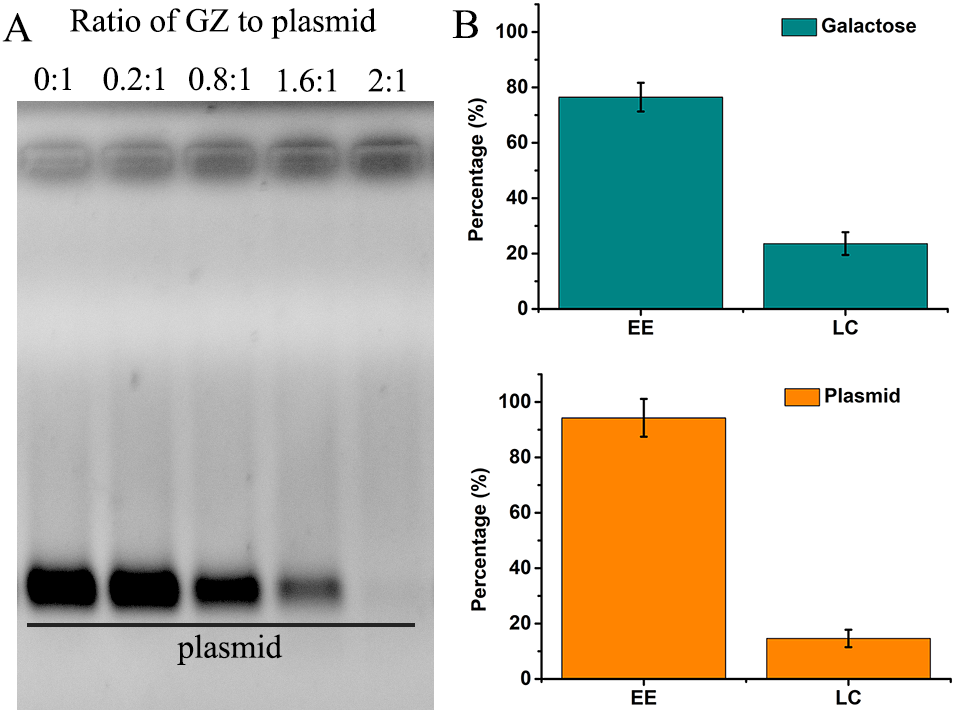


Figure S1 (A) The optimal ratio of GZ to plasmid was determined by agarose gel eletrophoresis. (B) The encapsulation efficiency and loading capacity of galactose and plasmid DNA in GA-RM/GZ/PL.


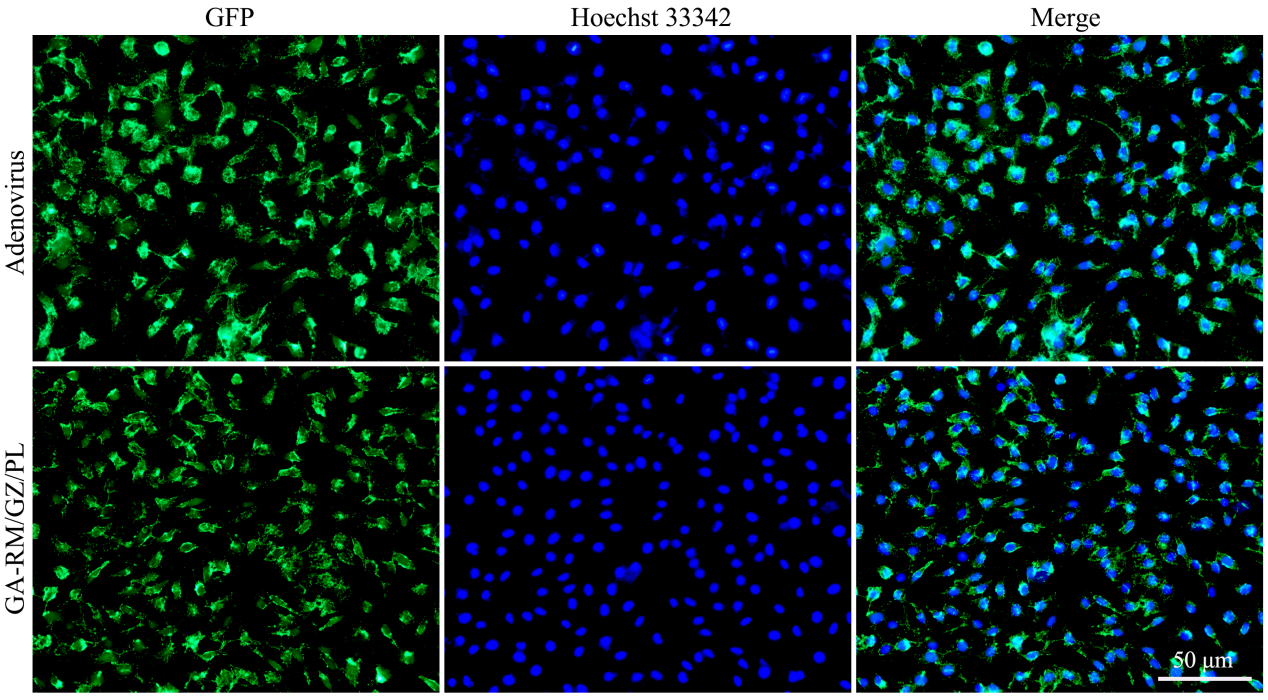


Figure S2 The comparation of transfection efficiency in GA-RM/GZ/PL and adenovirus treated hepatocytes.
